# Nuclear receptors in metabolic, inflammatory, and oncologic diseases: mechanisms, therapeutic advances, and future directions

**DOI:** 10.1186/s40001-025-03073-6

**Published:** 2025-09-09

**Authors:** Mohammed A. Abdel-Rasol, Wael M. El-Sayed

**Affiliations:** Department of Zoology, Faculty of Science, Ain Shams University, Abbassia, Cairo, 11566 Egypt

**Keywords:** Gene Therapy, Inflammation, MASLD, PPARs, Precision Medicine

## Abstract

Nuclear receptors (NRs) are a superfamily of ligand-activated transcription factors that regulate gene expression in response to metabolic, hormonal, and environmental signals. These receptors play a critical role in metabolic homeostasis, inflammation, immune function, and disease pathogenesis, positioning them as key therapeutic targets. This review explores the mechanistic roles of NRs such as PPARs, FXR, LXR, and thyroid hormone receptors (THRs) in regulating lipid and glucose metabolism, energy expenditure, cardiovascular health, and neurodegeneration. The therapeutic landscape for NRs has expanded with the approval of drugs like PPARγ agonists (pioglitazone, rosiglitazone) for diabetes, FXR agonists (obeticholic acid) for liver diseases, and selective TR agonists (resmetirom) for Metabolic dysfunction-Associated Steatohepatitis (MASH). However, challenges such as tissue-specific activation, drug resistance in chronic diseases, and potential carcinogenic risks continue to limit the full clinical efficacy of NR-targeted therapies. Emerging therapeutic strategies, including selective nuclear receptor modulators (SNRMs), dual and pan-NR agonists, and gene therapy approaches, aim to enhance receptor specificity while minimizing adverse effects. Furthermore, advances in artificial intelligence-driven drug discovery, CRISPR-based gene therapy, and microbiome-targeted interventions hold significant promise for refining the therapeutic efficacy and safety of NR-based treatments. A deeper understanding of NR crosstalk with metabolic, inflammatory, and oncogenic pathways will be crucial for developing next-generation therapies to overcome resistance mechanisms and improve clinical outcomes. These advancements, combined with precision medicine approaches, are poised to revolutionize NR-targeted therapies, offering more precise, effective, and safer treatments for a range of metabolic, inflammatory, and oncological diseases.

## Methodology

This narrative review synthesizes current evidence on the role of nuclear receptors (NRs) in metabolism and disease, as well as their therapeutic potential. To ensure comprehensive coverage, we searched PubMed for English-language articles published between 2000 and 2025 using keywords including “nuclear receptors,” “metabolism,” “inflammation,” “PPARs,” “FXR,” “LXR,” “thyroid hormone receptor,” “NR-targeted therapies,” and related terms. Studies focusing on key NRs such as PPARs, FXR, LXR, HNF4α, NR4A family, THRs, and VDR in the context of metabolic disorders, cancer, cardiovascular diseases, neurodegeneration, and inflammation were prioritized.

Relevant studies included preclinical research, clinical studies, and previous reviews that provided mechanistic insights, therapeutic developments, and discussions of emerging strategies such as selective nuclear receptor modulators, dual/pan-NR agonists, and gene therapy approaches. Articles without clear relevance to NR functions in disease contexts or lacking mechanistic insights were excluded.

While this review aimed to capture a broad and current perspective, it is limited by the exclusion of non-English studies and the reliance on available published data, which may introduce publication bias. The emphasis on preclinical and early clinical studies also means that some therapeutic strategies discussed are yet to be validated in large-scale human trials.

Despite these limitations, this narrative review aims to provide a clear, up-to-date synthesis of NR biology and therapeutic implications to guide future research and clinical translation.

## Introduction

Metabolic and chronic inflammatory diseases, including obesity, type 2 diabetes mellitus (T2DM), cardiovascular diseases (CVDs), and metabolic dysfunction-associated steatotic liver disease (MASLD), continue to pose a significant global health burden, with limited effective therapeutic options available to halt disease progression. There is an urgent need to identify and refine therapeutic strategies that can address the multifaceted pathophysiology of these conditions. Given their central role in regulating metabolic and inflammatory pathways, nuclear receptors (NRs) have emerged as promising therapeutic targets for these diseases. This review hypothesizes that strategically modulating NR activity offers a transformative approach for the treatment of metabolic and chronic inflammatory diseases while acknowledging the challenges related to specificity and resistance that must be addressed to maximize therapeutic efficacy.

NRs are a superfamily of ligand-activated transcription factors that regulate gene expression in response to small, lipophilic molecules, such as steroids, thyroid hormones, retinoids, bile acids, and fatty acids [1]. These receptors act as intracellular sensors, converting metabolic and hormonal signals into transcriptional changes that govern processes such as energy homeostasis, lipid and glucose metabolism, inflammation, immune responses, and cellular differentiation [2]. Unlike membrane-bound receptors, which rely on secondary messenger cascades, NRs directly bind to DNA at hormone response elements (HREs) in target gene promoters. Upon ligand binding, NRs undergo conformational changes, recruiting co-regulators and modifying chromatin to activate or repress transcription. Dysregulation of NR signaling has been linked to a variety of metabolic and chronic diseases, underscoring their crucial role in human health [3].

NRs are integral to the regulation of metabolic processes, including lipid metabolism, glucose homeostasis, mitochondrial function, energy expenditure, and inflammation. These functions position them as central players in the development of diseases such as obesity, insulin resistance, T2DM, metabolic dysfunction-associated fatty liver disease (MAFLD), and MASH. In addition, NRs are implicated in other chronic conditions, including CVDs (e.g., atherosclerosis and hypertension), neurodegenerative disorders (e.g., Alzheimer’s and Parkinson’s diseases), autoimmune diseases (e.g., rheumatoid arthritis), and various cancers (e.g., liver, breast, prostate, and colorectal cancers) [4, 5]. Given their central roles in metabolic and inflammatory pathways, NRs have emerged as promising therapeutic targets. Researchers are increasingly focused on developing drugs that modulate NR activity to restore metabolic balance and halt disease progression [4, 6].

Among the NRs involved in metabolism, peroxisome proliferator-activated receptors (PPARs) are particularly well studied. PPARs are key regulators of lipid metabolism [7], glucose homeostasis [8], and inflammation [9], and they function as heterodimers with retinoid X receptors (RXRs) to control important metabolic pathways [10]. PPARα, which is primarily expressed in the liver and skeletal muscle, promotes fatty acid oxidation and lipid clearance while controlling inflammation, making it a target for treating cardiovascular and liver diseases [11]. PPARγ, predominantly found in adipose tissue, enhances adipogenesis, improves insulin sensitivity, and regulates lipid storage, positioning it as a key therapeutic target for T2DM [12]. PPARδ, which is expressed ubiquitously, influences energy expenditure and mitochondrial function, playing a role in obesity and metabolic syndrome [13].

Another important NR in metabolism is the FXR, which acts as a bile acid sensor. FXR regulates cholesterol metabolism, bile acid synthesis, and lipid homeostasis, making it highly relevant in conditions like MASLD and MASH [14]. LXRs are also involved in regulating cholesterol homeostasis, modulating reverse cholesterol transport, and influencing inflammation and glucose metabolism, positioning them as promising targets for treating atherosclerosis and metabolic disorders [15].

Other NRs that contribute to metabolic regulation include the constitutive androstane receptor, which regulates xenobiotic metabolism, detoxification, and lipid metabolism [16], and RXRs, which serve as co-regulators influencing lipid and glucose metabolism, immune responses, and cell differentiation [17]. The vitamin D receptor (VDR), best known for its role in calcium and phosphate regulation, also impacts immune function and metabolic diseases, influencing insulin sensitivity and inflammatory responses [18]. The NR4A family (NR4A1/NUR77, NR4A2/NURR1, and NR4A3/NOR1) regulates mitochondrial function, energy metabolism, and inflammation, with implications in CVD, neurodegeneration, and cancer [19]. Hepatocyte nuclear factor 4 alpha (HNF4A) plays a crucial role in hepatic lipid metabolism and pancreatic β-cell function, with mutations in this gene linked to maturity-onset diabetes of the young (MODY) [20].

Given their involvement in these critical metabolic pathways, NRs are attractive targets for drug development. Several drugs targeting NRs have already been approved, and many others are currently under investigation. For instance, PPARγ agonists, such as thiazolidinediones (TZDs) like pioglitazone and rosiglitazone, have shown efficacy in improving insulin sensitivity in T2DM [21]. FXR agonists, such as obeticholic acid (OCA), are being explored for the treatment of MASLD, MASH, and cholestatic liver diseases [22]. LXR modulators are under investigation for managing atherosclerosis and lipid disorders [23]. VDR agonists, including calcitriol and paricalcitol, are used in chronic kidney disease and osteoporosis, while HNF4A modulators are being studied for diabetes and liver diseases [24].

Despite the promise of NR modulation, several challenges persist. One of the primary obstacles is tissue specificity. Many NR-targeting drugs affect a broad range of tissues, which can lead to unwanted side effects. For example, PPARγ agonists, while effective for insulin sensitivity, can induce fluid retention and weight gain [25]. Another issue is adaptive resistance, which may limit the long-term effectiveness of therapies, particularly in chronic diseases like metabolic syndrome [26]. To address these challenges, researchers are focused on developing SNRMs that target specific tissues or co-regulator interactions, minimizing systemic side effects. Additionally, combination therapies are being explored to enhance therapeutic outcomes, such as pairing NR agonists with other metabolic regulators like GLP-1 receptor agonists [27].

In conclusion, NRs are central regulators of lipid metabolism, glucose homeostasis, mitochondrial function, and inflammation, influencing the progression of diverse diseases from metabolic disorders to cancer. Their therapeutic potential is clear, but challenges such as tissue specificity, resistance, and off-target toxicity remain. This review outlines the current state of NR-targeted therapies, the barriers to clinical success, and strategies for improvement, including selective agonists, gene therapy, and precision medicine. Advances in pharmacology, molecular biology, and AI-driven drug discovery are poised to accelerate the development of safer, more effective NR-based treatments for metabolic and inflammatory diseases.

## Types of nuclear receptors

NRs are a diverse superfamily of ligand-activated transcription factors that regulate gene expression in response to various metabolic, hormonal, and environmental signals. Based on their mechanisms of action, ligand specificity, and subcellular localization, NRs can be categorized into four main types: Type I (steroid receptors), Type II (non-steroid receptors), Type III (orphan receptors), and Type IV (monomeric receptors). Each receptor type has a unique role in regulating critical physiological processes such as metabolic homeostasis, inflammation, endocrine signaling, and cell differentiation.

### Type I nuclear receptors

**Fig. 1:**
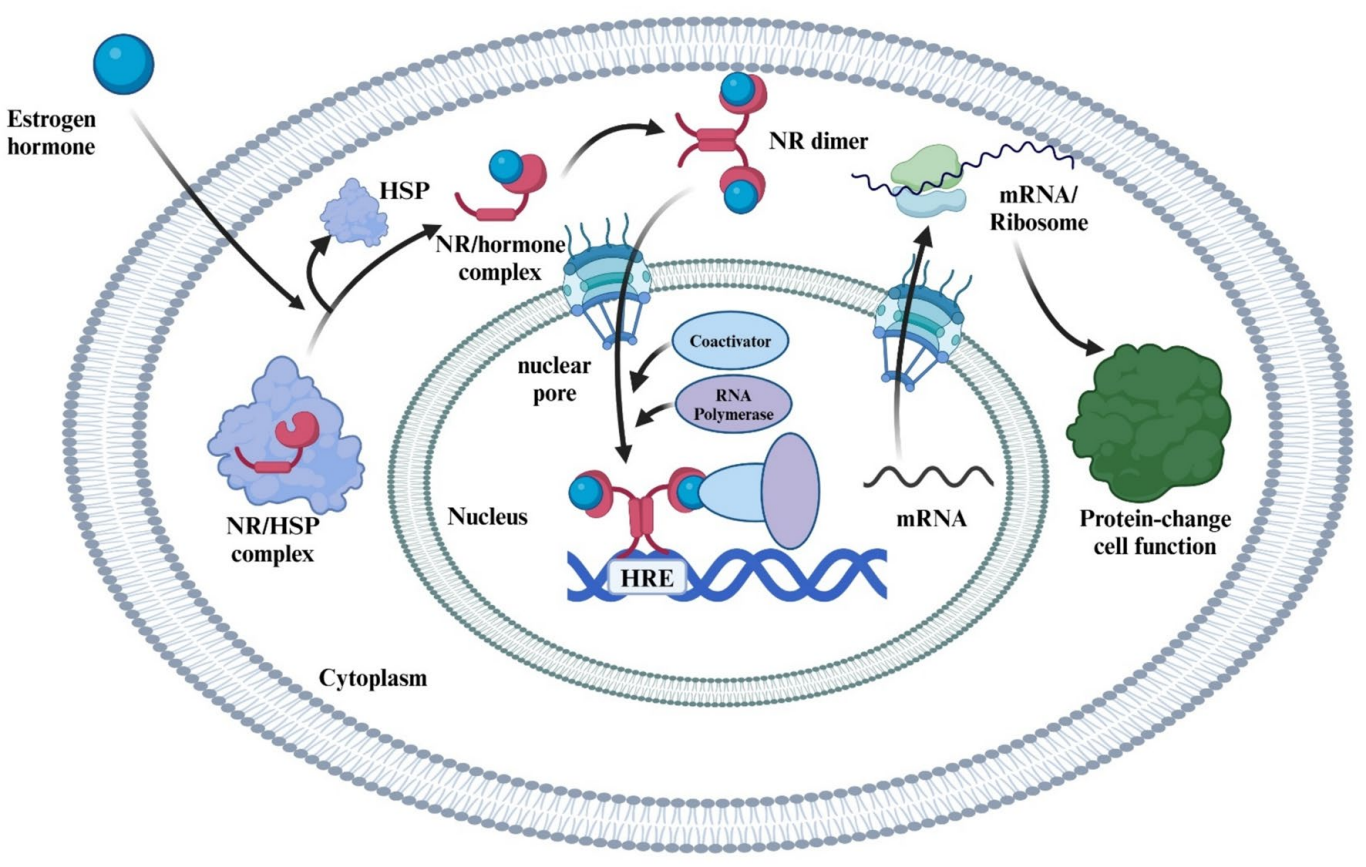
Mechanism of Action of Type I Nuclear Receptors in Endocrine Signaling. *NR* nuclear receptors, *HSP* heat shock protein, *HRE* hormone response element

Type I NRs, also known as steroid hormone receptors, play a central role in mediating endocrine signaling, metabolism, immune function, and the stress response. These receptors are typically localized in the cytoplasm in an inactive state, bound to heat shock proteins (HSPs) like HSP90 and HSP70, which stabilize them. Upon ligand binding, the receptors undergo a conformational change, dissociate from the chaperone proteins, and translocate to the nucleus. Once in the nucleus, the receptors bind to specific HREs on DNA (Fig. 1) to regulate the expression of target genes [28]. Additionally, post-translational modifications (PTMs), such as phosphorylation and ubiquitination, modulate receptor stability, activity, and subcellular localization, further influencing gene regulation. Dysregulation of Type I receptors is implicated in several diseases, including chronic inflammatory conditions, metabolic disorders, cancers, and neurodegenerative diseases, underscoring their potential as therapeutic targets [2].

#### Glucocorticoid receptor (GR)

The GR or NR3C1, activated by glucocorticoids like cortisol, remains associated with chaperone proteins such as HSP90, HSP70, FKBP51, and p23 in the cytoplasm when not bound to a ligand [29]. Upon ligand binding, GR dissociates from these chaperones, translocates to the nucleus, and binds to glucocorticoid response elements in DNA to modulate gene expression. GR can form homodimers or interact with other transcription factors, including NF-κB and AP-1, to fine-tune gene regulation [30]. GR plays a crucial role in inflammation suppression and regulates several metabolic processes, such as hepatic gluconeogenesis, lipolysis, and insulin sensitivity during chronic stress conditions [31]. Clinically, GR agonists such as dexamethasone and prednisone are widely used to treat autoimmune diseases, inflammatory disorders, and certain cancers [32]. Conversely, GR antagonists like mifepristone are being explored as potential treatments for Cushing’s syndrome and metabolic disorders [33]. While effective, GR-based therapies must be used with caution due to potential side effects, including immunosuppression, osteoporosis, and metabolic disturbances.

#### Estrogen receptor (ER)

The ER, activated by estrogens such as estradiol (E2), estrone (E1), and estriol (E3), exists in two isoforms—ERα and ERβ—which are differentially expressed across tissues [34]. Upon ligand binding, ER translocates to the nucleus, where it interacts with estrogen response elements (EREs) in DNA to regulate gene transcription [35]. In addition to genomic signaling, ER also participates in non-genomic signaling pathways by interacting with cytoplasmic kinases such as MAPK and PI3K/Akt [36]. Estrogens regulate multiple physiological processes, including reproductive physiology, bone density, cardiovascular health, and cognitive function. Clinically, selective estrogen receptor modulators (SERMs), such as tamoxifen and raloxifene, are used for breast cancer therapy and osteoporosis prevention [37]. Moreover, hormone replacement therapy (HRT) using estradiol is commonly prescribed to manage menopausal symptoms and support cardiovascular and skeletal health in postmenopausal women. Despite their benefits, the long-term use of ER modulators and HRT raises concerns about their potential to increase the risk of breast cancer and other hormone-dependent cancers, highlighting the need for more targeted and selective therapies.

#### Androgen receptor (AR)

The AR, activated by testosterone and its derivative dihydrotestosterone, remains bound to HSP90 in the cytoplasm in the absence of its ligands [38]. Upon ligand binding, AR dissociates from the chaperone, translocates to the nucleus, dimerizes, and binds to androgen response elements (AREs) in DNA to regulate gene expression [39]. AR is essential for male reproductive function, spermatogenesis, secondary sexual characteristics, and muscle and bone metabolism. Additionally, AR influences cognitive function and metabolic pathways. Clinically, AR agonists such as testosterone are used to treat hypogonadism and muscle wasting diseases [40], while AR antagonists like bicalutamide and enzalutamide are employed in the treatment of prostate cancer [41]. However, the use of AR-targeted therapies can lead to side effects such as cardiovascular problems and altered metabolism, necessitating careful management of treatment regimens.

#### Conclusion

In summary, Type I NRs, including the AR, GR, and ER, are central to regulating numerous physiological processes, such as hormone-driven metabolism, immune function, reproductive health, and stress adaptation. These receptors are integral to the pathogenesis of various diseases, including inflammatory disorders, metabolic dysfunctions, hormone-dependent cancers, and neurodegenerative conditions. Consequently, they represent critical therapeutic targets. Ongoing research is focused on improving the specificity of therapies targeting these receptors to minimize side effects and enhance therapeutic efficacy. Furthermore, exploring the interactions of Type I receptors with other signaling pathways will further advance the development of precision medicine strategies, which could lead to more effective and personalized treatments for these complex diseases.

### Type II nuclear receptors

**Fig. 2:**
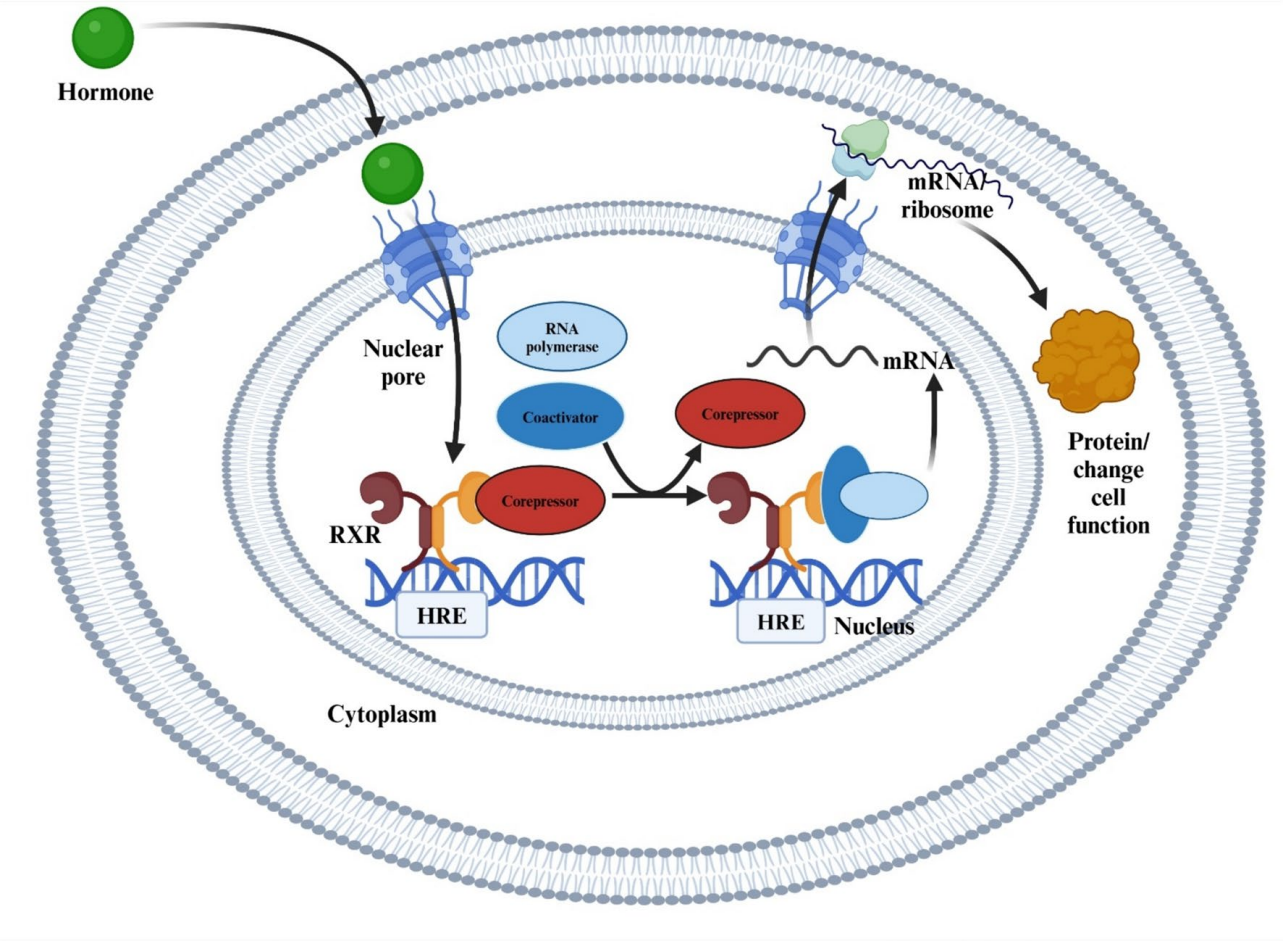
Mechanism of action of type II nuclear receptors in gene regulation. *RXR* retinoid X receptor, *HRE* hormone response element

Type II NRs differ from Type I receptors in that they are typically located in the nucleus, regardless of ligand binding. These receptors function as heterodimers with RXRs and bind directly to HREs on DNA to regulate transcription, even in the absence of ligands [42]. This unique mechanism allows them to maintain gene regulation with high precision (Fig. 2). Type II receptors play essential roles in metabolic homeostasis, development, differentiation, immune function, and neurogenesis, influencing energy metabolism, lipid regulation, and cognitive processes [43]. Their ability to interact with corepressors and coactivators further fine-tunes gene expression, which makes them central to both normal physiological processes and disease development. Dysregulation of these receptors is linked to metabolic disorders, neurodegeneration, cancer, and inflammatory diseases, emphasizing their importance as therapeutic targets [44].

#### Thyroid hormone receptor (THR)

The THR, activated by triiodothyronine (T3) and thyroxine (T4), is pivotal in regulating metabolism, growth, and development. There are two isoforms of THR: THRα and THRβ, each with distinct tissue-specific roles. THRα primarily influences heart rate and energy metabolism in the heart and skeletal muscle, while THRβ regulates cholesterol metabolism, thermogenesis, and cognitive functions in tissues such as the liver and brain [45]. In the absence of thyroid hormones, THRs associate with corepressors, which suppress gene transcription. Upon ligand binding, THRs undergo a conformational change, recruit coactivators, and activate gene transcription [46]. THR signaling is critical for regulating basal metabolic rate, mitochondrial biogenesis, lipid metabolism, and neuronal differentiation. This makes THR a potential therapeutic target for conditions like obesity, metabolic syndrome, and cognitive disorders. Clinical applications include THR agonists for hypothyroidism and metabolic disorders, while TR antagonists are being explored for thyroid hormone resistance syndromes and certain thyroid cancers [47]. However, managing THR-targeted therapies remains challenging due to potential side effects and the need for tissue-specific effects.

#### Retinoic acid receptor (RAR)

The retinoic acid receptor (RAR), activated by retinoic acid (a derivative of vitamin A), regulates several essential biological processes, including cell differentiation, embryonic development, immune function, and skin homeostasis. RAR exists in three isoforms—RARα, RARβ, and RARγ—each with distinct tissue distributions and roles: RARα is expressed in various tissues, RARβ plays a key role in neurogenesis and tumor suppression [48], and RARγ is vital for skin and bone development [49]. RARs bind to retinoic acid response elements (RAREs) in DNA in conjunction with RXR heterodimers. When unbound by ligand, RARs recruit corepressors to inhibit gene transcription; upon ligand binding, coactivators are recruited to activate transcription [50]. RARs are crucial for embryogenesis, skin differentiation, and immune function, and their activation can suppress inflammatory cytokines, which makes them potential targets for autoimmune diseases and inflammatory disorders. Clinically, RAR agonists like tretinoin and isotretinoin are used for treating acne and psoriasis, while RAR-targeting agents are employed in the treatment of acute promyelocytic leukemia to induce differentiation of leukemic cells [51].

#### Peroxisome proliferator-activated receptors (PPARs)

PPARs are Type II NRs that regulate lipid metabolism, glucose homeostasis, and inflammation. There are three primary isoforms of PPAR—PPARα, PPARγ, and PPARδ—each with distinct roles in metabolic regulation. PPARα, primarily expressed in the liver, heart, and muscle, promotes fatty acid oxidation and cholesterol metabolism, making it a target for treating dyslipidemia and CVDs [52]. PPARγ, found predominantly in adipose tissue, the pancreas, and immune cells, regulates adipogenesis and insulin sensitivity, positioning it as a key target for T2DM treatment [13]. PPARδ, expressed in muscles and the central nervous system, plays a role in mitochondrial biogenesis, fatty acid oxidation, and energy expenditure, offering promise for addressing obesity and neurodegenerative diseases [53]. PPARs bind to peroxisome proliferator response elements (PPREs) in DNA, forming heterodimers with RXRs to regulate lipid metabolism and inflammation [5]. Emerging research also suggests that PPARs may influence gut microbiota composition, which could have broader therapeutic implications [54]. Clinically, PPARγ agonists Such as TZDs Like pioglitazone and rosiglitazone are used to treat type 2 diabetes, while PPARα agonists like fibrates target hyperlipidemia and cardiovascular risks [55]. PPARδ agonists are currently under investigation for neuroprotection, muscle regeneration, and treating metabolic syndrome [56].

#### Conclusion

Type II NRs are integral in regulating metabolic processes, cell differentiation, neurodevelopment, and immune function. Their function as heterodimers with RXRs enables precise and dynamic gene regulation in response to hormonal signals, making them essential therapeutic targets for a range of diseases. With advancements in molecular biology, ongoing research is opening new avenues for targeting these receptors in the treatment of metabolic disorders, neurodegenerative diseases, and cancers. The potential for personalized medicine and targeted therapies remains high, though challenges in tissue-specific targeting and minimizing side effects must be addressed for optimal therapeutic outcomes.

### Type III nuclear receptors

Type III NRs, also known as orphan receptors, are unique in that their endogenous ligands are either unknown or exist at extremely low concentrations. Despite this, these receptors are crucial for regulating a wide range of physiological processes, including metabolism, steroidogenesis, immune function, neuroprotection, and cellular differentiation. Unlike ligand-dependent receptors, orphan receptors are often constitutively active or modulated by PTMs, protein–protein interactions, and environmental factors [57]. This flexibility in regulation allows orphan receptors to influence biological processes even in the absence of well-defined ligands. Their involvement in critical processes across multiple systems underscores their physiological and pathological relevance, making them promising targets for drug development—particularly in the treatment of metabolic disorders, neurodegenerative diseases, and cancer [58].

#### Liver receptor homolog-1 (LRH-1)

Liver receptor homolog-1 (LRH-1, NR5A2) is an orphan NR that plays a key role in regulating cholesterol metabolism, bile acid synthesis, steroidogenesis, and lipid homeostasis [59]. It is predominantly expressed in the liver, intestine, pancreas, and ovaries, where it regulates essential metabolic and reproductive functions. In the liver, LRH-1 controls genes involved in bile acid synthesis and cholesterol homeostasis, which are critical for lipid metabolism and glucose regulation. This makes LRH-1 an important factor in diseases such as MASLD and insulin resistance [60]. In the pancreas, LRH-1 promotes β-cell survival and insulin secretion, further linking it to diabetes pathophysiology [61]. Additionally, LRH-1 is involved in steroidogenesis and reproductive health, particularly in females, where it regulates estrogen and progesterone biosynthesis [62]. In the intestine, LRH-1 helps maintain stem cell renewal and gut barrier integrity, with activation shown to reduce inflammation in conditions like Crohn's disease and ulcerative colitis [63].

LRH-1 activity is modulated by PTMs such as SUMOylation and phosphorylation, which influence its stability and transcriptional activity [64]. Small-molecule LRH-1 agonists are currently being explored for their potential to regulate hepatic metabolism, improve bile acid homeostasis, and promote β-cell survival in diabetes [65]. However, challenges remain in identifying highly specific agonists that target LRH-1 without triggering undesirable off-target effects.

#### Nuclear receptor related 1 (NURR1)

NURR1 or NR4A2 is an orphan receptor primarily expressed in the central nervous system, especially in dopaminergic neurons. NURR1 plays a critical role in dopamine neuron survival, differentiation, and neurotransmission, which impacts motor function, cognition, and mood regulation. NURR1 maintains dopamine homeostasis by regulating genes involved in dopamine biosynthesis and neuroprotection. Dysfunction of NURR1 is strongly associated with neurodegenerative diseases like Parkinson’s disease (PD), where the degeneration of dopaminergic neurons leads to motor impairments [66]. NURR1 is also implicated in other neurological and psychiatric disorders, such as Alzheimer’s disease (AD), schizophrenia, and depression, through its regulation of inflammatory responses and neurotransmission [67].

Recent studies suggest that NURR1 could be a promising therapeutic target for neurodegeneration, with NURR1 agonists showing potential in preclinical models of PD by enhancing dopamine synthesis and reducing oxidative stress [2]. NURR1’s anti-inflammatory properties also make it a potential target for treating neuroinflammatory diseases such as multiple sclerosis [68]. As an orphan receptor, NURR1’s activity is regulated by protein–protein interactions and oxidative signaling, and pharmacological strategies—such as allosteric modulators and gene therapies—are being explored to harness its therapeutic potential [69]. However, challenges persist in developing drugs that can selectively activate or modulate NURR1 without adverse effects, particularly given its broad influence on inflammation and neurotransmission.

#### Conclusion

Type III NRs, such as NURR1 and liver receptor homolog-1 (LRH-1), are crucial regulators of metabolism, neuroprotection, immune modulation, and hormone synthesis. Their role in diverse physiological processes, despite the absence of well-defined ligands, underscores their potential as therapeutic targets. Advances in orphan receptor pharmacology and precision medicine are paving the way for new treatments for metabolic disorders, neurodegenerative diseases, and cancer. However, challenges in drug development, such as the identification of specific agonists and the modulation of receptor activity without off-target effects, remain key hurdles in realizing the full therapeutic potential of these receptors.

### Type IV nuclear receptors (monomeric receptors)

Type IV NRs, also known as monomeric receptors, are unique in that they function independently of RXR heterodimerization, distinguishing them from other NRs. This capability allows them to regulate essential biological processes directly as monomers, binding to specific DNA response elements (REs) to modulate gene expression. Key functions of these receptors include regulation of glucose and lipid metabolism, mitochondrial function, inflammation, neuroprotection, and cardiovascular health [70]. Their role as direct transcriptional regulators gives them precise control over metabolic and inflammatory homeostasis, which is crucial for maintaining cellular balance and preventing disease.

#### Hepatocyte nuclear factor 4 alpha (HNF4A)

HNF4A or NR2A1 is predominantly expressed in the liver, pancreas, kidneys, and intestines, where it regulates glucose metabolism, lipid homeostasis, and insulin secretion. In the liver, HNF4A controls genes involved in gluconeogenesis and lipid metabolism, which are essential for metabolic balance. In pancreatic β-cells, HNF4A plays a crucial role in insulin secretion and β-cell survival, making it an important factor in diabetes pathophysiology. Mutations in HNF4A are linked to MODY1, a condition marked by early-onset diabetes due to impaired insulin secretion [71]. Additionally, HNF4A maintains intestinal epithelial homeostasis, with dysfunction contributing to inflammatory bowel diseases (IBDs) like Crohn’s disease and ulcerative colitis [72]. PTMs such as SUMOylation and phosphorylation modulate HNF4A's activity, influencing its stability, DNA-binding affinity, and transcriptional activity [73].

Given its central role in metabolic regulation, HNF4A is a promising therapeutic target. Small-molecule agonists and gene therapies aimed at modulating HNF4A activity are being explored for the treatment of diabetes, MASLD, and IBD. However, targeting HNF4A remains challenging due to its widespread tissue expression and complex regulation, necessitating precise strategies to avoid unintended side effects.

#### NR4A family (NR4A1, NR4A2, NR4A3)

The NR4A family consists of three ligand-independent NRs: NR4A1 (NUR77), NR4A2 (NURR1), and NR4A3 (NOR1), which play crucial roles in inflammation, mitochondrial function, neurodegeneration, cardiovascular health, and metabolic regulation. These receptors act as immediate-early response genes, responding to cellular stress, inflammatory signals, and metabolic cues, and are regulated through PTMs and protein–protein interactions [74].

NR4A1 (NUR77) is involved in inflammation, mitochondrial biogenesis, and energy metabolism within immune cells, adipose tissue, and skeletal muscle. It has therapeutic potential for treating obesity and metabolic syndrome, and also influences cardiovascular health by regulating vascular smooth muscle cell proliferation [75]. NR4A2 (NURR1) is highly expressed in dopaminergic neurons, where it is essential for dopamine synthesis and neuronal survival. Dysfunction of NURR1 is linked to PD and neuroinflammation, prompting research into NURR1 agonists for treating neurodegenerative and psychiatric disorders [66]. NR4A3 (NOR1) regulates skeletal muscle adaptation, mitochondrial biogenesis, and metabolic homeostasis, with implications for enhancing endurance performance and addressing metabolic conditions such as insulin resistance, heart disease, and hypertension [76].

The NR4A family is regulated by several signaling pathways, including AMPK, mTOR, and PI3K/Akt, which integrate signals related to nutrient availability, inflammation, and cellular stress [77]. Therapeutic strategies targeting these receptors, including small-molecule agonists, gene therapy, and CRISPR-based modulation, are being explored for metabolic diseases, neurodegenerative disorders, and autoimmune diseases [78].

#### Conclusion

Type IV NRs, including HNF4A and the NR4A family, are pivotal in regulating key physiological processes such as glucose metabolism, lipid homeostasis, neuroprotection, cardiovascular function, and immune modulation. Their unique ability to function as monomers and bind directly to DNA response elements enables them to regulate gene expression with precision. As central players in metabolic, inflammatory, and neurodegenerative diseases, these receptors present significant therapeutic potential. Ongoing research into gene therapies, small-molecule agonists, and other molecular interventions offers promising avenues for the treatment of metabolic diseases, neurodegenerative disorders, and cardiovascular conditions.

## Mechanism of action of nuclear receptors

NRs are ligand-activated transcription factors that regulate gene expression through direct DNA interaction. They play essential roles in cell differentiation, metabolism, immune responses, and development.

The mechanism of NR action begins with ligand binding to the receptor’s ligand-binding domain, inducing conformational changes. This leads to the dissociation of corepressors and recruitment of coactivators, such as SRC-1, CBP/p300, and PGC-1α, enabling receptor binding to HREs in gene promoters and initiating transcription [79].

NR-mediated gene regulation depends on a dynamic balance between coactivators and corepressors. Coactivators like the SRC family and p300/CBP enhance gene expression by promoting histone acetylation and chromatin relaxation, allowing RNA polymerase access to promoters [80]. In contrast, corepressors such as NCoR, SMRT, and HDACs suppress transcription by deacetylating histones, compacting chromatin, and reducing transcriptional accessibility [81]. This regulation is finely tuned by ligand binding, receptor isoforms, chromatin context, and ligand–receptor interactions.

In addition, NRs engage chromatin remodeling complexes, such as SWI/SNF, to reposition nucleosomes and expose gene promoters for transcription. They also recruit histone acetyltransferases (HATs) or histone deacetylases (HDACs) to modulate chromatin accessibility, a critical step in transcriptional regulation [82]. Epigenetic modifications, including SUMOylation, phosphorylation, and methylation, further influence NR activity by affecting DNA-binding affinity and transcriptional potential [83]. Technologies like single-cell ATAC-seq and ChIP-seq have advanced understanding of chromatin accessibility and NR function across tissues and disease states [84].

NRs also interact with key kinase signaling pathways, including MAPK, PI3K/Akt, and mTOR, to integrate metabolic cues and stress signals. For example, the MAPK pathway phosphorylates ER and PPARγ, enabling ligand-independent activation linked to breast and prostate cancer resistance [85, 86]. The PI3K/Akt pathway regulates FOXO, PPARγ, and GR, impacting glucose homeostasis and immune function [87]. Akt-mediated phosphorylation of HNF4A influences pancreatic β-cell function and diabetes pathophysiology [88]. The mTOR pathway, which governs nutrient sensing and protein synthesis, interacts with NRs like PPARδ and LXR to regulate lipid metabolism and mitochondrial biogenesis [89]. Dysregulation of these pathways can contribute to disease, as seen in mTOR-driven hyperactivation of ERα in estrogen-positive breast cancer [90].

### Clinical and therapeutic implications

Understanding NR signaling and its interactions with chromatin remodeling and kinase pathways has opened new therapeutic avenues for diseases such as cancer, metabolic disorders, and neurodegenerative conditions. One notable advancement is the development of SNRMs, which can enhance receptor activity with greater tissue specificity, minimizing off-target effects.

Another promising approach is the development of allosteric modulators and degrader technologies, such as Proteolysis-Targeting Chimeras. These technologies allow for the selective degradation of dysregulated NRs, offering potential treatments for hormone-driven cancers like ER-positive breast cancer [91], AR-driven prostate cancer [92], and metabolic diseases such as T2DM [93].

Epigenetic therapies targeting NR-coactivator interactions are also being explored, particularly in cancers resistant to hormone-based treatments. These therapies aim to reverse resistance by targeting specific phosphorylation sites or chromatin regulators, restoring sensitivity to endocrine therapies [94].

The integration of NR biology with advanced omics technologies—such as RNA-seq, ATAC-seq, and metabolomics—enables the development of personalized treatments tailored to an individual’s metabolic and genomic profile. This approach holds significant promise for precision medicine, offering targeted interventions for a wide range of conditions.

Recent advances in understanding NR-ligand specificity, epigenetic modifications, and post-translational regulation have paved the way for innovative therapeutic interventions. Future breakthroughs in drug discovery, NR engineering, and CRISPR-based gene editing offer exciting prospects for restoring metabolic homeostasis and addressing disease progression, providing new hope for patients with conditions linked to NR dysfunction [95].

## Nuclear receptors in metabolic disorders, cancer progression, and neurodegeneration

### Peroxisome proliferator-activated receptor alpha (PPARα)

PPARα is a key NR predominantly expressed in the liver, skeletal muscle, heart, and kidney. It regulates crucial processes such as fatty acid oxidation, lipid metabolism, and anti-inflammatory signaling. Activated by ligands like long-chain fatty acids and fibrates, PPARα induces the transcription of genes involved in β-oxidation and lipoprotein metabolism, including CPT1, ACOX1, and PGC-1α [96].

#### Metabolic regulation

The activation of PPARα plays a central role in regulating lipid metabolism and reducing triglyceride accumulation in the liver, thus contributing to the prevention of hepatic steatosis and conditions such as MASLD and hyperlipidemia [97]. PPARα acts as a transcription factor that regulates the expression of genes involved in fatty acid oxidation, lipoprotein metabolism, and lipid transport. Upon activation, PPARα enhances the expression of genes such as CPT1, ACOX1, and medium-chain acyl-CoA dehydrogenase (MCAD), which are involved in β-oxidation and fatty acid transport into the mitochondria for energy production. These processes promote fatty acid catabolism, thereby reducing hepatic triglyceride accumulation and mitigating the development of MASLD.

Fibrates, such as fenofibrate and bezafibrate, act as PPARα agonists, enhancing fatty acid uptake and β-oxidation while improving lipoprotein profiles. Fibrates activate PPARα, leading to the upregulation of genes that promote the breakdown of fatty acids and the synthesis of lipoproteins, including apolipoprotein A1 (APOA1) and apolipoprotein B (APOB). These changes result in the reduction of circulating triglycerides, the lowering of VLDL levels, and the increase in HDL -cholesterol. The net effect is a favorable shift in lipid profiles, which helps mitigate the risk of atherosclerosis and related CVDs [98].

These metabolic effects are further supported by AMPK signaling, which is activated under conditions of energy stress and works in concert with PPARα to enhance fatty acid oxidation and inhibit lipogenesis. Additionally, SIRT1, a deacetylase involved in energy metabolism, interacts with PPARα to increase mitochondrial biogenesis and enhance the oxidative capacity of tissues, further supporting lipid metabolism regulation [99].

In conclusion, PPARα activation plays a vital role in regulating lipid metabolism by promoting fatty acid oxidation, improving lipoprotein metabolism, and reducing triglyceride accumulation, thus preventing the onset of conditions like MASLD and hyperlipidemia. Fibrates and other PPARα agonists can further enhance these beneficial effects, improving lipid profiles and reducing the risk of CVD.

#### Interaction with the gut microbiome

PPARα plays a pivotal role in linking metabolism with the gut microbiome. It regulates bile acid synthesis, secretion, and reabsorption in the liver, processes essential for fat digestion and absorption. By activating genes such as CYP7A1, which encodes the rate-limiting enzyme in bile acid synthesis, and BSEP, which regulates bile acid transport, PPARα maintains the enterohepatic circulation of bile acids, supporting lipid homeostasis in the liver and gut [100].

The gut microbiome, in turn, influences PPARα activity through its metabolites. Short-chain fatty acids (SCFAs) like butyrate, acetate, and propionate activate PPARα via GPR41/43 on intestinal epithelial cells, promoting fatty acid oxidation in the liver and enhancing bile acid metabolism. This creates a positive feedback loop between the microbiome and liver metabolism. Additionally, SCFAs activate AMPK and SIRT1 pathways, further supporting lipid metabolism and energy balance [100].

Dysbiosis disrupts this interaction, impairing PPARα activation and bile acid metabolism while reducing SCFA production. These disruptions contribute to metabolic diseases such as MASLD and obesity. Altered bile acid synthesis and composition can reduce fat digestion and nutrient absorption, worsening metabolic disturbances. Dysbiosis can also increase inflammatory cytokines like TNF-α and IL-6, which are linked to insulin resistance and metabolic dysfunction [101].

In summary, PPARα is a crucial mediator between lipid metabolism and the gut microbiome, regulating bile acid metabolism and maintaining energy balance. Disruptions in PPARα signaling or microbiome function can impair this balance, contributing to MASLD, obesity, and related metabolic disorders.

#### Anti-inflammatory properties

PPARα is widely recognized for its anti-inflammatory effects, which are crucial for cardiovascular health. PPARα exerts its anti-inflammatory effects primarily by suppressing pro-inflammatory transcription factors such as NF-κB and AP-1. Upon activation, PPARα interacts with these transcription factors to inhibit their nuclear translocation and the subsequent transcription of pro-inflammatory cytokines like TNF-α and IL-6. By decreasing the production of these cytokines, PPARα helps limit chronic inflammation, which is a key factor in atherogenesis and the development of CVD [102].

Moreover, PPARα reduces the expression of adhesion molecules Such as vascular cell adhesion molecule 1 (VCAM-1) and intercellular adhesion molecule 1 (ICAM-1), which are involved in the adhesion of monocytes to the endothelium. This process is critical in the formation of foam cells, a hallmark event in the development of atherosclerotic plaques. By inhibiting these adhesion molecules, PPARα helps reduce the accumulation of lipid-laden cells in blood vessels, thus preventing the progression of atherosclerosis [103].

In terms of vascular protection, PPARα promotes endothelial health by modulating NO production and vascular reactivity. Through AMPK and eNOS pathways, PPARα activation enhances vasodilation and blood flow, reducing the risk of vascular dysfunction that often accompanies inflammatory states. This effect supports the prevention of cardiovascular events, particularly in conditions like metabolic syndrome and diabetic dyslipidemia, where inflammation plays a central role [103].

Fibrates, such as fenofibrate and bezafibrate, act as PPARα agonists and have shown promising results in reducing cardiovascular events in patients with metabolic syndrome and diabetic dyslipidemia. These drugs promote lipid metabolism, decrease triglyceride levels, and increase HDL cholesterol, all of which contribute to improved cardiovascular health. However, the long-term benefits of fibrates on cardiovascular outcomes remain under investigation [104].

The current research is also focusing on the development of dual and pan-PPAR agonists (PPARα/δ agonists), which aim to enhance both lipid metabolism and vascular protection while minimizing potential side effects. These dual agonists could offer improved therapeutic potential by targeting multiple PPAR isoforms, thus providing broader benefits for both metabolic and cardiovascular health [105].

In conclusion, PPARα plays a crucial role in reducing inflammation and providing vascular protection. Its effects on pro-inflammatory cytokines, adhesion molecules, and vascular reactivity highlight its significance in CVD prevention. The development of PPARα agonists and dual PPAR agonists offers promising strategies for treating CVD and metabolic disorders while improving patient outcomes.

#### Therapeutic implications

Given PPARα’s roles in lipid metabolism, inflammation, and metabolic regulation, it presents a promising therapeutic target for treating metabolic disorders such as hyperlipidemia, MASLD, and CVDs. Fibrates, like fenofibrate and bezafibrate, have been used for decades to modulate PPARα activity, improving lipid profiles and reducing cardiovascular risks in patients with metabolic syndrome. However, the clinical application of fibrates has limitations, particularly concerning their long-term benefits and side effect profiles [104].

Emerging research is exploring novel therapies that target PPARα more specifically. For instance, dual PPARα/δ agonists are being developed to enhance lipid metabolism and improve cardiovascular protection with fewer side effects than traditional fibrates. Furthermore, therapies targeting the PPARα–gut microbiome axis may offer innovative approaches for treating metabolic diseases, given the growing evidence of the gut microbiome's influence on metabolic health.

In conclusion, PPARα’s regulation of fatty acid oxidation, lipid metabolism, and inflammation positions it as a critical player in maintaining metabolic homeostasis. Continued research into selective agonists, combination therapies, and microbiome interactions could provide new avenues for treating a wide range of metabolic and cardiovascular disorders.

### Peroxisome proliferator-activated receptor gamma (PPARγ)

PPARγ is a critical NR primarily expressed in adipose tissue, liver, pancreas, and immune cells. It plays a vital role in regulating metabolism, immune modulation, inflammation, and cell differentiation, making it a crucial therapeutic target for conditions like T2DM, cancer, and neurodegenerative diseases.

#### Metabolic regulation and glucose homeostasis

PPARγ is essential for regulating adipogenesis, glucose metabolism, and insulin sensitivity. PPARγ promotes lipid storage and glucose uptake by enhancing the expression of genes such as FABP4, GLUT4, and adiponectin in adipocytes and skeletal muscle [106]. Through these mechanisms, PPARγ facilitates the proper storage of lipids in adipose tissue, preventing ectopic fat accumulation, which is a major contributor to insulin resistance. The activation of PPARγ in pre-adipocytes promotes their differentiation into mature adipocytes, thereby improving lipid storage capacity and reducing the ectopic deposition of fat in muscles and liver, which is closely linked to the development of T2DM [107].

In addition to its role in adipogenesis, PPARγ also modulates systemic inflammation by reducing the expression of pro-inflammatory cytokines such as TNF-α and IL-6 and promoting the secretion of anti-inflammatory adipokines like adiponectin. Adiponectin is crucial for mitigating chronic inflammation, which is often associated with T2DM and insulin resistance. PPARγ regulates the expression of adiponectin through the PI3K/Akt and AMPK signaling pathways, which are involved in glucose uptake and fatty acid oxidation, contributing to improved insulin sensitivity and reduced chronic inflammation in adipose tissue and skeletal muscle [107].

Furthermore, the activation of PPARγ has beneficial effects on endothelial function and oxidative stress, both of which are critical for preventing cardiovascular complications, which are common in T2DM patients. PPARγ activation improves eNOS expression through AMPK and SIRT1 pathways, promoting nitric oxide production and improving vascular reactivity. By reducing oxidative stress through nuclear factor erythroid 2-related factor 2 (Nrf2) pathway activation, PPARγ helps preserve endothelial function, reducing the risk of atherosclerosis and CVDs [108].

Through these multiple mechanisms—lipid metabolism, glucose regulation, insulin sensitivity, and inflammation—PPARγ remains a critical target for managing metabolic diseases, particularly T2DM. Drugs like TZDs, which act as PPARγ agonists, are used to improve insulin sensitivity and promote glucose uptake in tissues, further emphasizing the importance of PPARγ in metabolic disease management.

#### Immune modulation and inflammation

Beyond its critical roles in metabolism, PPARγ is a powerful modulator of immune responses. PPARγ exerts its anti-inflammatory effects primarily by suppressing NF-κB signaling. NF-κB is a key transcription factor involved in the production of pro-inflammatory cytokines such as TNF-α, IL-6, and IL-1β, which are elevated in conditions like obesity and insulin resistance [109]. Upon activation, PPARγ interacts with the IκB kinase (IKK) complex, inhibiting NF-κB activation, thus reducing the transcription of genes involved in macrophage infiltration and cytokine production. This suppression of NF-κB signaling helps mitigate the chronic inflammation that is commonly associated with metabolic disorders such as T2DM.

In addition to inhibiting NF-κB signaling, PPARγ also shifts the polarization of macrophages toward an anti-inflammatory phenotype. Activation of PPARγ induces the expression of M2 macrophage markers, such as CD163 and arginase 1 (Arg1), which are associated with tissue repair and immune tolerance. This shift promotes a reduction in inflammation within adipose tissue and other key metabolic organs like the liver and muscles, improving insulin sensitivity and promoting metabolic health [110]. The M2 macrophages in adipose tissue play a pivotal role in limiting the inflammatory response by secreting anti-inflammatory cytokines such as IL-10 and TGF-β, further promoting tissue homeostasis and reducing the risk of insulin resistance and T2D.

These immune-modulatory effects of PPARγ suggest that it is not only a crucial player in managing metabolic disorders but also a promising therapeutic target for inflammation-driven conditions. PPARγ agonists, such as TZDs, are already used in clinical practice to improve insulin sensitivity and regulate macrophage activity. These drugs may also have potential in treating autoimmune diseases and chronic inflammatory conditions, where inflammation plays a central role in disease progression [109, 110]. Thus, PPARγ is a key regulator of both metabolic health and immune homeostasis, making it an important therapeutic target in a variety of inflammation-related diseases.

#### Oncology applications

Recent studies have highlighted PPARγ's potential role in cancer, particularly in colorectal and breast cancers. Activation of PPARγ exerts anti-proliferative, pro-apoptotic, and anti-inflammatory effects by inhibiting cancer-promoting pathways such as the Wnt/β-catenin and modulating estrogen receptor (ER) signaling [87]. Wnt/β-catenin is a key signaling pathway that regulates cell proliferation and survival, often overactive in various cancers. PPARγ activation suppresses this pathway by promoting the degradation of β-catenin through increased expression of Axin and glycogen synthase kinase 3 beta (GSK-3β), both of which regulate β-catenin stability and translocation. This results in cell cycle arrest, limiting tumor growth and inhibiting cancer progression.

Furthermore, PPARγ activation influences ER signaling, particularly in breast cancer. It enhances Erα expression and function, thereby inhibiting estrogen-mediated cell proliferation in ER-positive breast cancers. This modulation of ER signaling helps limit tumor growth and resistance to hormonal therapies, offering a potential therapeutic advantage in ER-positive breast cancer [111].

Additionally, PPARγ ligands, including TZDs, omega-3 fatty acids, and curcumin, have demonstrated anti-tumor activity. These ligands work by modulating immune cell function in the tumor microenvironment, reducing pro-tumorigenic inflammation, and enhancing anti-tumor immunity. PPARγ activation enhances the function of anti-tumor immune cells, such as CD8 + T cells and natural killer (NK) cells, by promoting the secretion of anti-inflammatory cytokines like IL-10 and TGF-β in tumor-associated macrophages [112]. This results in reduced tumor-associated inflammation and improved immune surveillance, further limiting tumor growth and spread.

In summary, the activation of PPARγ exerts beneficial effects in cancer by inhibiting pro-cancerous pathways like Wnt/β-catenin, modulating ER signaling, and improving immune responses within the tumor microenvironment. PPARγ agonists hold significant promise as novel therapeutic agents for cancers, particularly those associated with chronic inflammation and immune dysfunction, such as colorectal cancer and breast cancer.

#### Neurodegeneration and Alzheimer’s disease

In neurodegenerative diseases, including AD, PPARγ is being investigated for its neuroprotective potential. Chronic activation of microglia and astrocytes triggers neuroinflammation, which exacerbates the production of pro-inflammatory cytokines such as TNF-α, IL-6, and IL-1β, as well as the deposition of amyloid-beta (Aβ) plaques, key contributors to AD pathology [113]. PPARγ activation helps suppress these pro-inflammatory cytokines by inhibiting the NF-κB pathway and by modulating the expression of M2 macrophage markers like CD163 and Arg1, promoting the polarization of microglia toward an anti-inflammatory M2 phenotype [114]. The M2 microglia phenotype is associated with tissue repair, immune tolerance, and the suppression of neuroinflammation, which aids in reducing cognitive decline associated with AD.

Furthermore, PPARγ activation enhances the clearance of Aβ through the regulation of Aβ transporters Like LDL receptor-related protein 1 (LRP) and ATP-binding cassette Subfamily A member 1 (ABCA1). This helps reduce Aβ plaque deposition in the brain, a hallmark feature of AD pathology. PPARγ-induced clearance of Aβ is mediated through microglial activation, which is stimulated by pathways like PPARγ/AMPK signaling, promoting autophagy and lysosomal degradation of Aβ aggregates [113, 114].

Clinical trials with PPARγ agonists, such as pioglitazone and rosiglitazone, have shown mixed results in improving cognitive function in early-stage AD patients, likely due to variability in patient subtypes and the stage of disease progression [115]. The activation of PPARγ in the brain may also result in metabolic side effects, such as weight gain and fluid retention, which can be problematic in the long-term treatment of neurodegenerative diseases.

To address the limitations of current therapies, research is focusing on developing selective PPARγ modulators (SPPARMs). These molecules aim to retain the neuroprotective effects of PPARγ while minimizing its metabolic side effects by selectively targeting specific PPARγ isoforms involved in neuroprotection. The development of SPPARMs could offer an opportunity to improve the safety and efficacy of PPARγ-targeted treatments for AD patients [116].

Moreover, combining PPARγ agonists with other therapies, such as anti- Aβ treatments or glucagon-like peptide-1 (GLP-1) receptor agonists, is being explored to improve both metabolic function and cognitive outcomes in AD patients. GLP-1 receptor agonists, which also exhibit neuroprotective properties, may complement PPARγ activation to provide a synergistic effect, enhancing both insulin sensitivity and neuroprotection in the brain [117].

#### Conclusion

In conclusion, PPARγ plays a pivotal role in regulating metabolism, immune modulation, inflammation, and cell differentiation. These functions make it a crucial therapeutic target for managing conditions like T2DM, cancer, and neurodegenerative diseases. While current PPARγ agonists, such as TZDs, provide therapeutic benefits, there is growing interest in developing SPPARMs and combination therapies. These approaches aim to maximize the receptor's therapeutic potential while minimizing side effects. Ongoing research into these novel therapies offers exciting prospects for more effective and safer treatment strategies in the future (Table 1).

**Table 1:** Exploring the functions and therapeutic potential of PPARα and PPARγ receptors in metabolic health

Feature	PPARα	PPARγ
Primary expression sites	Liver, skeletal muscle, heart, kidney	Adipose tissue, liver, pancreas, immune cells
Main functions	Fatty acid oxidation, lipid metabolism, anti-inflammatory signaling	Regulates adipogenesis, glucose metabolism, insulin sensitivity, immune modulation
Activation mechanisms	Activated by long-chain fatty acids and fibrates	Activated by TZDs, omega-3 fatty acids, and curcumin
Target genes/proteins	CPT1, ACOX1, PGC-1α, APOA1, APOB	FABP4, GLUT4, adiponectin, M2 macrophages
Metabolic role	Regulates lipid metabolism, prevents NAFLD, hyperlipidemia, cardiovascular diseases	Regulates glucose metabolism, improves insulin sensitivity, reduces ectopic fat
Anti-inflammatory role	Suppresses pro-inflammatory cytokines (TNF-α, IL-6), reduces endothelial dysfunction	Suppresses NF-kB signaling, modulates macrophage polarization (M2 anti-inflammatory)
Therapeutic applications	Fibrates (fenofibrate, bezafibrate) for lipid metabolism and cardiovascular disease management	TZDs for type 2 diabetes, potential use in cancer and neurodegenerative diseases
Diseases/conditions targeted	NAFLD, hyperlipidemia, cardiovascular disease	Type 2 diabetes, obesity, cancer, neurodegenerative diseases (e.g., Alzheimer's)

*PPARα* Peroxisome proliferator-activated receptor alpha, *PPARγ* peroxisome proliferator-activated receptor gamma, *NAFLD* non-alcoholic fatty liver disease, *TZDs* thiazolidinediones, *CPT1* carnitine palmitoyltransferase 1, *ACOX1* Acyl-CoA Oxidase 1, *PGC-1α* peroxisome proliferator-activated receptor gamma coactivator 1 alpha, *APOA1* Apolipoprotein A1, *APOB* Apolipoprotein B, *FABP4* fatty acid binding protein 4, *GLUT4* glucose transporter type 4, *TNF-α* Tumor Necrosis Factor alpha, *IL-6* Interleukin-6

### Farnesoid X receptor (FXR)

The FXR is a bile acid-activated NR that plays a central role in maintaining bile acid homeostasis, regulating cholesterol metabolism, lipid levels, and controlling inflammation. FXR’s influence spans various biological processes, particularly in the liver, gut, cardiovascular system, and even the brain. By modulating bile acid production and metabolism, FXR is critical for maintaining metabolic health and preventing liver diseases, such as cholestasis and gallstone formation [118].

#### Metabolic regulation and glucose homeostasis

FXR plays a profound role in hepatic lipid metabolism and glucose regulation, particularly through the gut–liver axis. Activation of FXR triggers the production of fibroblast growth factor 19, which acts on the liver to inhibit gluconeogenesis and promote glycogen storage, thus enhancing insulin sensitivity [119]. FGF19 binds to its receptor fibroblast growth factor receptor 4 (FGFR4) on hepatocytes, activating the MAPK and PI3K/Akt pathways, which contribute to reduced glucose production and increased glycogen storage. This process improves overall metabolic health by reducing hepatic fat accumulation, regulating lipid metabolism, and protecting the liver from inflammation and fibrosis.

Additionally, FXR activation Suppresses the transcription factor sterol regulatory element-binding protein 1c (SREBP-1c), which plays a critical role in lipogenesis. SREBP-1c regulates the synthesis of fatty acids and triglycerides, and its downregulation by FXR activation helps prevent excessive lipid accumulation in the liver, promoting the clearance of VLDL and reducing the risk of fatty liver disease [22]. FXR also influences the expression of ABCA1 and lipoprotein lipase (LPL), both of which are involved in lipoprotein metabolism, contributing to VLDL and LDL clearance. Through these mechanisms, FXR activation helps maintain lipid homeostasis and prevent dyslipidemia, which is crucial for protecting the liver from inflammation and fibrosis.

FXR agonists, such as OCA, are being actively investigated as potential treatments for conditions like MASH and Primary Biliary Cholangitis (PBC) [120]. OCA enhances FXR activation, leading to improved glycogen storage, reduced glucose production, and better lipid clearance, making it a promising therapeutic option. However, prolonged activation of FXR can lead to dyslipidemia, characterized by increased LDL cholesterol and triglycerides, as well as pruritus, a common side effect shared by most FXR agonists [121]. These side effects pose a challenge to its broader therapeutic use, highlights the need for further research to optimize FXR-targeted therapies that balance metabolic benefits with potential side effects.

In conclusion, FXR activation plays a critical role in glucose metabolism, lipid regulation, and the protection of the liver from inflammation and fibrosis. FXR agonists, such as OCA, are not only effective in treating MASH and PBC but have also been FDA approved, making them established therapeutic options. While OCA demonstrates clear efficacy, challenges such as dyslipidemia and pruritus—a common side effect shared by most FXR agonists—need to be addressed for broader therapeutic applications.

#### FXR in gut microbiome regulation, inflammation, and fibrosis

FXR not only regulates hepatic lipid metabolism but also plays a significant role in modulating the gut microbiome through its influence on bile acid metabolism. Activation of FXR promotes the synthesis of bile acids, which function as signaling molecules that affect the diversity and composition of the microbiota in the gut. FXR activation leads to the upregulation of genes involved in bile acid synthesis, such as CYP7A1 and BSEP, which help regulate the enterohepatic circulation of bile acids. Bile acids, in turn, serve as ligands for FXR and TGR5, modulating the gut microbiota’s composition and influencing intestinal homeostasis [122].

In diseases like MASLD and MAFLD, dysbiosis can disrupt bile acid homeostasis, exacerbate hepatic lipid accumulation, and promote inflammation. Altered conversion of primary bile acids (like chenodeoxycholic acid) to secondary bile acids (such as deoxycholic acid) can shift the gut microbiome toward pro-inflammatory species while reducing beneficial, anti-inflammatory populations. These changes can worsen insulin resistance and liver inflammation, which are key features of MASLD and MAFLD [120].

Secondary bile acids also influence intestinal tight junction integrity and microbial fermentation pathways. Disruption of these processes can increase intestinal permeability, allowing endotoxins such as LPS to cross into the portal circulation. LPS activates NF-κB signaling in the liver, triggering pro-inflammatory cytokine production, oxidative stress, and hepatocellular injury [122].

Beyond these microbiome-mediated mechanisms, FXR exerts direct anti-inflammatory effects. By inhibiting NF-κB signaling, FXR reduces transcription of pro-inflammatory cytokines (TNF-α, IL-1β, IL-6) and chemokines, limiting immune cell infiltration and tissue damage. FXR also regulates acute-phase proteins such as CRP, contributing to the control of hepatic and systemic inflammation [123].

FXR activation further provides anti-fibrotic benefits by inhibiting HSC activation, lowering α-SMA expression, and modulating the TGF-β pathway. Downregulation of TGF-β1 reduces extracellular matrix production and collagen deposition, slowing fibrosis progression. FXR agonists, such as OCA, have been shown to suppress α-SMA expression, decrease pro-fibrotic mediator levels, and reduce collagen synthesis. Both experimental and clinical studies have demonstrated that OCA treatment can improve fibrosis scores, attenuate inflammatory activity, and enhance metabolic parameters [124].

By restoring bile acid homeostasis, improving gut barrier function, and directly modulating inflammatory and fibrotic pathways, FXR agonists like OCA target both the triggers and downstream consequences of chronic liver injury [122]. This integrated action underscores the therapeutic potential of FXR activation in conditions such as MASLD, MAFLD, MASH, and PBC, while ongoing research focuses on maximizing efficacy and minimizing adverse effects.

#### Vascular health and inflammation control

FXR’s influence extends to vascular health, where it plays a crucial role in reducing inflammation. By inhibiting NF-κB signaling, FXR helps reduce the expression of adhesion molecules like VCAM-1 and ICAM-1, which are involved in vascular inflammation and atherogenesis [125]. These actions preserve endothelial integrity and prevent vascular dysfunction, which can otherwise contribute to CVDs.

However, when FXR activity is antagonized, vascular inflammation increases, leading to cholesterol accumulation and endothelial dysfunction. Studies in FXR-deficient mice have demonstrated a higher susceptibility to atherosclerosis due to impaired lipid metabolism and enhanced inflammation [126]. Additionally, FXR modulates endothelial function by regulating eNOS activity and influencing arterial stiffness. Thus, FXR's role in controlling vascular inflammation is crucial for maintaining cardiovascular health.

#### Brain function and neuroinflammation

Emerging research has highlighted FXR's potential role in brain function, particularly in regulating brain lipid metabolism and neuroinflammation. Dysregulated bile acid signaling can cross the BBB, contributing to neurodegenerative diseases by impairing neuronal function, synaptic plasticity, and promoting neuroinflammation. The disruption of bile acid homeostasis can lead to an imbalance in primary and secondary bile acids, which act as signaling molecules within the brain, affecting various neuroinflammatory pathways. Activation of FXR in the brain has been shown to regulate the synthesis and conversion of bile acids, and it can directly influence neuroinflammation and synaptic plasticity through the NF-κB and AMPK signaling pathways [127].

In preclinical models of AD and vascular dementia, FXR agonists have demonstrated the ability to enhance synaptic plasticity, reduce cognitive decline, and mitigate neuroinflammation. FXR activation in the hippocampus and other regions of the brain promotes the expression of brain-derived neurotrophic factor (BDNF) and other neurotrophic factors through cAMP response element-binding protein (CREB) signaling. BDNF plays a critical role in synaptic remodeling and neurogenesis, enhancing neuronal survival and plasticity, which are crucial for learning and memory processes. The suppression of neuroinflammation is also facilitated by the FXR-induced inhibition of NF-κB signaling, which typically promotes the production of pro-inflammatory cytokines like TNF-α, IL-6, and IL-1β in microglia and astrocytes. These cytokines are key contributors to the chronic inflammation observed in neurodegenerative diseases like AD [128].

FXR activation also promotes mitochondrial health in the brain by regulating mitochondrial biogenesis through PGC-1α and SIRT1 pathways. This supports neuronal energy production, which is essential for maintaining synaptic function and protecting against oxidative stress, a hallmark of neurodegeneration. AMPK activation through FXR signaling further enhances neuronal energy metabolism, optimizing glucose utilization and fatty acid oxidation in the brain [128].

These findings suggest that FXR modulation may offer therapeutic benefits not only in metabolic diseases but also in neurodegenerative conditions. By influencing brain lipid metabolism, enhancing synaptic plasticity, and reducing neuroinflammation, FXR agonists could potentially play a role in protecting the brain from neurodegeneration. Therefore, further exploration of FXR's neuroprotective effects could lead to the development of novel strategies for treating AD and other neurodegenerative disorders.

#### Conclusion

FXR is a critical regulator of lipid metabolism, synaptic plasticity, and neuroinflammation, playing a pivotal role in brain health. Its multifaceted functions in bile acid metabolism, vascular health, and inflammation position FXR as a promising therapeutic target for metabolic, cardiovascular, and neurodegenerative diseases. While FXR agonists like OCA show promise in treating liver diseases such as MASH and PBC, managing potential dyslipidemia from prolonged activation remains a key challenge. Ongoing research into selective FXR modulators (sFXRMs), dual agonists, and the gut–brain axis will be essential for developing safer, more effective therapeutic strategies to harness FXR’s full potential in treating complex diseases.

### Liver X receptor (LXR)

The LXR, consisting of LXRα and LXRβ, are NRs activated by oxysterols and act as cholesterol sensors that regulate cholesterol metabolism, lipid homeostasis, glucose metabolism, and inflammation. LXRα is predominantly expressed in the liver, adipose tissue, and macrophages, while LXRβ is more widely distributed in various tissues, including the brain, heart, and kidney. Both receptors work together to maintain lipid balance and have important roles in cardiovascular health and metabolic regulation.

LXR activation promotes cholesterol efflux through transporters such as ABCA1 and ABCG1, enhancing reverse cholesterol transport and reducing the formation of foam cells—key factors in the development of atherosclerosis [129]. Despite these beneficial effects, LXR activation also induces lipogenesis, which can lead to increased triglyceride production and hepatic steatosis. This dual role presents challenges for the use of LXR-targeted therapies in treating metabolic disorders like T2DM and MASLD.

#### Role in cholesterol metabolism and lipid regulation

LXR activation plays a pivotal role in cholesterol metabolism by promoting the clearance of cholesterol through HDL-mediated reverse cholesterol transport. This process helps prevent cholesterol accumulation in the arterial walls, thereby reducing the risk of atherosclerosis [130]. LXR activation induces the expression of ABCA1 and ABC-G1, both of which are involved in the efflux of cholesterol from peripheral tissues to the liver and then to HDL particles. The HDL particles transport excess cholesterol to the liver, where it can be excreted in the bile or converted into bile acids, helping maintain cholesterol homeostasis and reduce cardiovascular risk.

However, while LXR activation is beneficial for cardiovascular protection, it also stimulates lipogenesis through the upregulation of SREBP-1c, a transcription factor that controls the expression of genes involved in triglyceride production in the liver [131]. SREBP-1c activation increases the synthesis of fatty acids and triglycerides, contributing to hepatic steatosis when this process becomes excessive. In conditions like T2DM and MASLD, this dual effect of LXR activation complicates the development of LXR-targeted therapies. While it reduces cholesterol buildup, it simultaneously promotes lipid accumulation, which exacerbates liver fat deposition and insulin resistance.

The balance between cholesterol clearance and lipogenesis is regulated by several pathways. In the liver, LXR activation leads to increased expression of genes involved in cholesterol efflux and bile acid synthesis, such as CYP7A1 and BSEP. Additionally, LXR also activates PPARα, which regulates fatty acid oxidation and lipid metabolism, thus potentially reducing the risk of hepatic steatosis. However, the stimulation of SREBP-1c leads to increased lipogenesis and triglyceride accumulation, contributing to hepatic lipid overload.

This dual effect of LXR activation presents challenges for the development of LXR agonists for diseases like T2DM and MASLD, where both cholesterol homeostasis and lipid accumulation are critical concerns. Researchers are now focusing on developing selective LXR modulators that can enhance the beneficial effects of cholesterol clearance without inducing excessive lipogenesis [131].

In conclusion, LXR activation plays a critical role in maintaining cholesterol homeostasis and lipid metabolism, promoting reverse cholesterol transport via HDL and influencing lipogenesis through SREBP-1c. This dual action complicates the therapeutic targeting of LXR in diseases like T2DM and MASLD, where both cholesterol accumulation and hepatic lipid deposition need to be carefully managed.

#### Anti-inflammatory effects

LXRs exert significant anti-inflammatory effects, particularly in the context of cardiovascular health. LXR activation suppresses the production of pro-inflammatory cytokines such as TNF-α, IL-6, and IL-1β, which are critical drivers of inflammation in the arterial walls and other tissues. By inhibiting these cytokines, LXR activation helps reduce the formation of foam cells in atherosclerotic plaques, a key event in the progression of atherosclerosis [132]. This process involves the uptake of oxidized LDL by macrophages, which leads to foam cell formation and plaque development. LXR activation reduces foam cell formation by inducing the expression of ABCA1 and ABCG1, promoting cholesterol efflux from macrophages and reducing their lipid accumulation.

Moreover, LXR activation inhibits NF-κB signaling, a central regulator of inflammation. NF-κB is activated by pro-inflammatory stimuli such as TNF-α and IL-1β, leading to the transcription of various pro-inflammatory genes in endothelial cells and macrophages. LXR reduces the activation of NF-κB by promoting the expression of IκB kinase alpha (IKBα), which binds to and sequesters NF-κB dimers, preventing their nuclear translocation and transcriptional activity. This reduces the expression of pro-inflammatory genes, including VCAM-1 and ICAM-1, which are involved in leukocyte adhesion and migration into inflamed tissues [131, 132].

LXR activation also regulates NO production, which is essential for maintaining endothelial function and blood pressure regulation. NO, produced by eNOS, has vasodilatory effects and helps maintain vascular health by reducing vascular inflammation and oxidative stress. LXR activation upregulates eNOS expression through the AMPK and PPARγ pathways, thereby increasing NO production and improving vascular reactivity and blood pressure regulation [131].

These anti-inflammatory effects make LXRs a promising therapeutic target for atherosclerosis and other inflammatory diseases. By reducing inflammation in the vascular walls, promoting cholesterol efflux, and improving endothelial function, LXR activation could provide significant benefits for managing CVDs and other chronic inflammatory conditions [132].

In conclusion, LXR activation provides a multifaceted anti-inflammatory effect by reducing pro-inflammatory cytokine production, inhibiting NF-κB signaling, improving cholesterol efflux, and enhancing NO production. These mechanisms collectively make LXRs a potential therapeutic target for CVDs and inflammatory disorders, emphasizing their importance in vascular health and immune regulation.

#### Glucose metabolism and insulin sensitivity

LXRs are crucial in regulating glucose homeostasis and improving insulin sensitivity. LXR activation enhances insulin sensitivity by promoting the expression of GLUT4, a key protein involved in glucose uptake in muscle and adipose tissue. GLUT4 translocation to the cell membrane is facilitated by AMPK signaling, which is activated upon LXR engagement. This pathway enhances glucose uptake, particularly after meals, helping maintain blood glucose levels within a healthy range [133].

In the liver, LXR activation reduces hepatic gluconeogenesis—the production of glucose from non-carbohydrate precursors like amino acids and lactate. This effect is primarily mediated through the downregulation of SREBP-1c, which is a key transcription factor involved in promoting glucose production. By inhibiting SREBP-1c, LXR reduces the expression of key enzymes involved in gluconeogenesis, such as PEPCK and G6Pase, thus lowering hepatic glucose output [133].

These mechanisms make LXR activation particularly relevant for managing T2DM, as it helps regulate glucose levels and improves insulin sensitivity. However, careful modulation of LXR activity is necessary to avoid exacerbating lipid accumulation, especially in the liver. LXR activation also stimulates lipogenesis by inducing SREBP-1c, which can lead to increased triglyceride production in the liver, a contributing factor to hepatic steatosis and insulin resistance.

In addition to its effects on glucose metabolism, LXR signaling influences pancreatic β-cell function. LXR activation can enhance β-cell Survival and Function by modulating the expression of genes involved in insulin biosynthesis and secretion, Such as insulin 1 (Ins1) and insulin 2. LXR agonists have been shown to promote β-cell proliferation and reduce β-cell apoptosis, suggesting potential therapeutic applications for improving glucose control and insulin production in individuals with T2DM [133].

In conclusion, LXR activation plays a key role in glucose metabolism and insulin sensitivity by promoting GLUT4 expression, reducing hepatic gluconeogenesis, and improving β-cell function. However, its dual effect on lipid metabolism necessitates careful modulation to avoid complications Such as Hepatic steatosis. The therapeutic potential of LXR agonists for treating type 2 diabetes and improving glucose control is promising, but it requires further optimization to balance the benefits and risks associated with lipid accumulation.

#### Interaction with the gut–liver axis

The gut–liver axis plays a crucial role in regulating LXR activity. Microbiome-derived metabolites, such as bile acids and SCFAs, significantly influence LXR-driven metabolic effects. Bile acids, which act as both LXR ligands and regulators of lipid metabolism, help maintain cholesterol homeostasis, modulate hepatic lipid storage, and regulate inflammation. Upon activation by bile acids, LXR regulates genes involved in cholesterol efflux, such as ABCA1 and ABCG1, which help transfer excess cholesterol from tissues into the liver and promote its conversion to bile acids. This process helps regulate lipid metabolism and prevents cholesterol accumulation in the liver and arterial walls, ultimately reducing the risk of atherosclerosis [134].

Furthermore, LXR activation plays a key role in regulating hepatic lipid storage by influencing the expression of lipogenic genes such as SREBP-1c, which promotes fatty acid synthesis. However, dysregulated LXR activation can exacerbate hepatic steatosis, particularly in the context of dysbiosis. Dysbiosis can alter bile acid profiles, leading to impaired LXR activity and disruption of the gut–liver axis, which contributes to the development of metabolic disorders such as MASLD and insulin resistance [135].

In addition to bile acids, SCFAs, such as butyrate, acetate, and propionate, produced by gut bacteria during fiber fermentation, influence LXR activity. SCFAs act through GPR41/43 on gut epithelial cells, and their signaling can activate AMPK and PPARγ, which are involved in promoting lipid metabolism and glucose regulation. SCFAs also modulate inflammation by enhancing the production of anti-inflammatory cytokines such as IL-10 and TGF-β, contributing to a reduction in systemic inflammation and supporting liver function.

The interplay between the microbiome and bile acid metabolism highlights the critical role of LXR in maintaining liver function and metabolic health. Disruptions in this axis can impair lipid and cholesterol metabolism, leading to the progression of MASLD, insulin resistance, and atherosclerosis. As a result, modulation of the microbiome, potentially through the use of probiotics or prebiotics, may provide new therapeutic opportunities to regulate LXR activity and improve outcomes in metabolic diseases. For example, probiotics that promote the growth of beneficial gut bacteria may help restore bile acid balance, improve LXR activation, and reduce hepatic inflammation, offering a promising approach for treating metabolic disorders [134, 135].

#### Conclusion

In conclusion, LXRs are integral to regulating cholesterol metabolism, lipid homeostasis, glucose metabolism, and inflammation. LXR activation promotes cholesterol clearance, reduces the risk of atherosclerosis, and exerts anti-inflammatory effects, making them promising targets for treating CVDs. However, the stimulation of lipogenesis complicates their use in managing metabolic disorders such as T2DM and MASLD. Additionally, LXRs enhance insulin sensitivity and influence β-cell function, presenting potential benefits for diabetes management. The gut–liver axis also plays a significant role in modulating LXR activity, with bile acids and SCFAs acting as key modulators. This interaction highlights the potential of microbiome-targeted therapies in regulating LXR activity and improving metabolic health.

### Hepatocyte nuclear factor 4 alpha (HNF4α)

HNF4α is a critical NR and transcription factor that plays a central role in regulating hepatic glucose metabolism, lipid processing, insulin secretion, and inflammatory responses. It is essential for maintaining hepatic insulin sensitivity, pancreatic β-cell function, and the regulation of various metabolic pathways. HNF4α coordinates the expression of genes involved in glucose transport, glycogen storage, and β-oxidation, making it integral to both liver and pancreatic function.

#### Role in glucose homeostasis and insulin secretion

HNF4α is a nuclear transcription factor essential for regulating glucose homeostasis and insulin secretion. In the liver, HNF4α modulates the expression of key genes involved in gluconeogenesis, including G6Pase and PEPCK, which are crucial for the production of glucose from non-carbohydrate sources. This regulation occurs through direct binding of HNF4α to promoter regions of these genes, activating their transcription and ensuring adequate glucose output during fasting or metabolic stress. The expression of these genes is also influenced by cAMP/PKA signaling and CREB, with HNF4α acting downstream to integrate hormonal and nutrient signals [136].

In the pancreas, HNF4α plays a pivotal role in β-cell function and insulin secretion. It regulates the transcription of the INS gene, GLUT2, and glucokinase (GCK)—all vital for glucose sensing and insulin production in pancreatic β-cells. GLUT2 facilitates glucose entry into β-cells, and GCK acts as a glucose sensor, phosphorylating glucose to initiate insulin secretion. HNF4α enhances their transcription by interacting with Pancreatic and duodenal homeobox 1 and the HNF1α-HNF4α transcriptional network, which coordinates gene expression critical for glucose-stimulated insulin release [137].

Mutations in HNF4α are associated with MODY1, a monogenic form of diabetes characterized by β-cell dysfunction, impaired insulin secretion, and progressive hyperglycemia. These mutations disrupt HNF4α’s DNA-binding capacity or coactivator recruitment, impairing the regulation of insulin gene expression and glucose metabolism. Additionally, HNF4α activity is modulated by the AMPK and PI3K/Akt pathways, linking it to broader metabolic signaling networks that influence hepatic glucose output and insulin action [138].

In summary, HNF4α is central to maintaining glucose homeostasis by regulating hepatic gluconeogenesis through G6Pase and PEPCK and promoting insulin secretion in β-cells via INS, GLUT2, and GCK expression. Disruption of these pathways due to HNF4α mutations leads to metabolic dysfunctions such as MODY1, emphasizing its therapeutic relevance in genetic and metabolic forms of diabetes.

#### Liver dysfunction and MASLD/MASH

HNF4α is a master transcription factor critical for maintaining hepatic insulin sensitivity, lipid processing, and glucose metabolism, and its dysregulation is a major contributor to the pathogenesis of MASLD and MASH [139]. HNF4α regulates genes such as APOA1, APOB, MTTP, and CYP7A1, which are essential for lipid export, fatty acid β-oxidation, and bile acid synthesis, thereby preventing hepatic steatosis. Reduced HNF4α expression impairs these pathways, resulting in triglyceride accumulation, glucose intolerance, and hepatic insulin resistance, all of which contribute to MASLD progression [140]. Beyond metabolic regulation, HNF4α also exerts anti-inflammatory and anti-fibrotic effects by suppressing pro-inflammatory cytokines like TNF-α and IL-6, and fibrogenic markers such as TGF-β and COL1A1, largely through inhibition of TGF-β/Smad signaling, which limits hepatic stellate cell (HSC) activation and extracellular matrix deposition [177]. HNF4α cooperates with co-regulators like PGC-1α, which stimulates mitochondrial biogenesis and PPARα-mediated fatty acid oxidation, and FOXA2, which governs lipid transport and gluconeogenesis, further amplifying its metabolic control [141].

Moreover, PTMs such as SUMOylation and phosphorylation under conditions of oxidative stress diminish HNF4α’s nuclear localization and transcriptional activity, worsening inflammation and insulin resistance. Epigenetically, HNF4α activity is modulated by HATs like CBP/p300, which promote transcription via histone acetylation, and HDACs, which repress its function by inducing chromatin condensation [142].

Given its central role in metabolic and inflammatory regulation, therapeutic strategies targeting HNF4α—such as gene therapy, small-molecule activators, PTM modulators, and epigenetic therapies—are being actively investigated to restore liver homeostasis and halt MASLD/MASH progression.

#### Interaction with the gut–liver axis

HNF4α also plays a significant role in regulating the gut–liver axis, where changes in gut microbiota composition can influence its metabolic pathways and contribute to liver inflammation and lipid accumulation. The gut microbiome produces metabolites that interact with HNF4α, impacting gene expression related to lipid metabolism and inflammation [141]. Dysbiosis—imbalance in the gut microbiome—can disrupt HNF4α-mediated metabolic processes, further contributing to the development of metabolic diseases like MASLD and insulin resistance.

Recent studies have suggested that modulating HNF4α activity through dietary interventions or microbiome modulation might offer a promising strategy for restoring metabolic balance, reducing liver inflammation, and improving lipid homeostasis. This highlights the potential for gut–liver axis-targeted treatments in managing metabolic disorders.

#### Role in liver and pancreatic cancer

HNF4α is a key transcription factor involved in liver development, metabolic regulation, and pancreatic β-cell function, but its dysregulation is implicated in the progression of several cancers, notably hepatocellular carcinoma (HCC) and pancreatic ductal adenocarcinoma (PDAC). In HCC, the loss of HNF4α disrupts epithelial identity and promotes epithelial-to-mesenchymal transition (EMT), a process that enhances cancer cell invasiveness, proliferation, chemoresistance, and metastatic potential. Mechanistically, this occurs through the downregulation of epithelial markers like E-cadherin and upregulation of mesenchymal markers such as N-cadherin and vimentin, driven in part by activation of the Wnt/β-catenin and TGF-β/Smad pathways [143]. Loss of HNF4α removes transcriptional repression of EMT-related genes and disrupts normal hepatic differentiation, which is strongly associated with poor prognosis in HCC patients.

In contrast, in PDAC, HNF4α can exhibit context-dependent oncogenic activity. In certain subtypes, particularly those with classical differentiation, overexpression of HNF4α supports tumor progression by promoting metabolic reprogramming—notably enhancing lipogenesis and glycolysis through the SREBP-1c and mTOR pathways. These metabolic shifts increase tumor cell survival and proliferation. HNF4α also contributes to chemoresistance in PDAC via modulation of xenobiotic metabolism genes and detoxifying enzymes, which are regulated through NRF2 and PXR crosstalk [144].

Furthermore, HNF4α’s dual role—acting as a tumor suppressor in HCC and a contextual oncogene in PDAC—is mediated through its interaction with co-factors such as PGC-1α, FOXA2, and SOX9, as well as epigenetic modifications that affect its chromatin-binding activity. For instance, aberrant histone deacetylation by HDACs in liver cancer suppresses HNF4α transcriptional activity, whereas in PDAC, enhanced HNF4α binding to oncogenic loci drives malignancy [144].

Due to this dual function, selective HNF4α modulators are under investigation as potential cancer therapies. These modulators aim to fine-tune HNF4α activity—restoring its tumor-suppressive function in HCC while limiting its tumor-promoting role in PDAC. Strategies include context-specific small-molecule inhibitors or activators, and epigenetic therapies that reprogram HNF4α target gene expression depending on cancer subtype and cellular environment.

#### Conclusion

HNF4α plays a critical role in regulating metabolic processes, inflammation, and disease progression, especially in cancers like HCC and PDAC, as well as metabolic disorders such as MASLD, MASH, and diabetes. Its dysregulation can drive tumor progression and chemoresistance, while its loss promotes HCC, and overexpression in certain PDAC subtypes enhances tumor aggressiveness. Targeting HNF4α through selective agonists or modulators presents a promising strategy to restore its function in metabolic diseases and provide targeted treatments for specific cancer subtypes. Ongoing research into HNF4α modulators offers exciting potential for improving cancer therapies and metabolic health (Table 2).

**Table 2:** A comparative overview of FXR, LXR, and HNF4α receptors: roles in metabolism, inflammation, and Disease

Feature		FXR		LXR		HNF4α
Primary expression sites	Liver, gut, cardiovascular system, brain		Liver, adipose tissue, macrophages (LXRα); brain, heart, kidney (LXRβ)		Liver, pancreas, gut	
Main functions	Bile acid homeostasis, cholesterol metabolism, lipid regulation, inflammation control		Cholesterol metabolism, lipid homeostasis, glucose metabolism, inflammation regulation		Regulates glucose metabolism, lipid processing, insulin secretion, inflammatory responses	
Activation mechanisms	Activated by bile acids (e.g., chenodeoxycholic acid), FXR agonists (e.g., OCA)		Activated by oxysterols, LXR agonists		Activated by metabolic signals, co-regulators (e.g., PGC-1α), transcriptional factors	
Target genes/proteins	FGF19, CYP7A1, BSEP, ABCA1, LPL		ABCA1, ABCG1, SREBP-1c		G6Pase, PEPCK, GLUT2, INS, APOA1, APOB	
Metabolic role	Regulates glucose metabolism, lipid metabolism, protects liver from inflammation and fibrosis		Promotes reverse cholesterol transport, reduces foam cells, regulates glucose metabolism		Regulates hepatic glucose production, insulin secretion, lipid processing, prevents NAFLD	
Anti-inflammatory role	Inhibits NF-kB signaling, reduces vascular inflammation, modulates eNOS activity		Inhibits TNF-α, IL-6, IL-1β, modulates NF-kB signaling, enhances NO production		Suppresses pro-inflammatory cytokines (e.g., TNF-α, IL-6), inhibits TGF-β/Smad signaling	
Therapeutic applications	OCA for NASH, PBC, liver diseases		LXR agonists for atherosclerosis, Type 2 diabetes, cardiovascular diseases		Gene therapy, small-molecule activators, epigenetic therapies for NAFLD, NASH, diabetes	
Diseases/conditions targeted	NAFLD, MAFLD, NASH, cholestasis, atherosclerosis		Type 2 diabetes, NAFLD, atherosclerosis, cardiovascular diseases		NAFLD, NASH, MODY1, liver cancer, pancreatic cancer	

*FXR* Farnesoid X receptor, *LXR* liver X receptor, *HNF4α* Hepatocyte nuclear factor 4 alpha, *NASH* non-alcoholic steatohepatitis, *PBC* primary biliary cholangitis, *NAFLD* non-alcoholic fatty liver disease, *MAFLD* metabolic associated fatty liver disease, *MODY1* Maturity-onset diabetes of the young type 1, *FGF19* fibroblast growth factor 19, *CYP7A1* cytochrome P450 family 7 Subfamily a member 1, *BSEP* bile salt export pump, *ABCA1* ATP-binding cassette Subfamily a member 1, *LPL* lipoprotein lipase, *SREBP*-1c: sterol regulatory element-binding protein 1c, *TNF-α* tumor necrosis factor alpha, *IL-6* Interleukin 6, *eNOS* endothelial nitric oxide synthase, *NO* nitric oxide, *PGC-1α* peroxisome proliferator-activated receptor gamma coactivator 1 alpha

### Thyroid hormone receptor (THR)

#### Glucose homeostasis and insulin secretion

THRs are critical in regulating glucose metabolism in peripheral tissues such as the liver, muscle, adipose tissue, and pancreas. THRs function as nuclear transcription factors, binding to thyroid hormone response elements (TREs) in DNA to regulate genes involved in glucose production, storage, and utilization. In the liver, THRs enhance the transcription of key gluconeogenic enzymes such as PEPCK, G6Pase, and F1,6-BPase through activation of the AMPK and sirtuin 1 (SIRT1) pathways, which are critical for glucose production from non-carbohydrate precursors like amino acids and lactate during fasting or increased energy demand [145]. This process ensures glucose is available to maintain euglycemia under energy-deprived conditions.

In muscle and adipose tissue, THRs improve insulin sensitivity by increasing insulin receptor (IR) expression and enhancing GLUT4 translocation to the plasma membrane through PI3K/Akt signaling, facilitating glucose uptake and regulation of blood glucose levels [146]. THRα plays a pivotal role in the pancreas, where it supports insulin secretion by modulating insulin gene expression through CREB and calcium/calmodulin-dependent protein kinase II (CaMKII) pathways. THRα activation increases insulin release in response to glucose fluctuations, ensuring timely insulin production and release during meals or periods of elevated blood glucose [147].

THR signaling also modulates insulin sensitivity by improving the function of insulin receptor substrates (IRS), such as IRS1 and IRS2, in peripheral tissues. Through MAPK and PI3K/Akt pathways, THRs enhance glucose uptake and promote insulin responsiveness. However, thyroid dysfunction can disrupt this balance. In hypothyroidism, reduced THR activity impairs glucose production and insulin sensitivity, contributing to hyperglycemia and insulin resistance [148]. In contrast, hyperthyroidism increases THR activity, leading to excessive gluconeogenesis and glucose intolerance, despite enhanced insulin secretion, through mechanisms involving AMPK and SIRT1 signaling pathways [149].

Targeting THR signaling with THR agonists or antagonists holds potential for treating Type 1 and T2DM by enhancing insulin sensitivity and glucose production regulation without systemic thyroid hormone side effects. For instance, THRβ agonists may selectively enhance insulin sensitivity in peripheral tissues and regulate hepatic glucose production [150].

In conclusion, THRs are vital for maintaining glucose homeostasis, and understanding their mechanisms offers promising therapeutic strategies for managing diabetes and metabolic syndrome.

#### Energy metabolism, thermogenesis, and mitochondrial function

THRs are pivotal regulators of energy metabolism, thermogenesis, and mitochondrial Function. By promoting the expression of uncoupling protein 1 (UCP1) in brown adipose tissue (BAT), THRs facilitate thermogenesis, a process that dissipates energy as heat, thus playing a significant role in energy balance and weight regulation [151]. THRα and THRβ mediate UCP1 expression through activation of the AMPK pathway, which promotes fatty acid oxidation and thermogenic processes, ensuring the body can efficiently utilize energy stored in adipose tissue for heat production, especially in cold environments or during physical activity [149].

Furthermore, THRs enhance mitochondrial efficiency and ATP production, which are essential for muscle performance and endurance. THRβ in particular regulates mitochondrial function by promoting the expression of key genes involved in mitochondrial biogenesis, such as PPARα and PGC-1α [152]. These genes coordinate mitochondrial replication and the production of the electron transport chain components, thereby improving ATP production and enhancing the oxidative capacity of muscle cells. Additionally, THRα plays a role in supporting muscle function by regulating myosin heavy chain expression, contributing to muscle contraction and endurance during prolonged activity.

These functions are further supported by interactions with critical signaling pathways like AMPK and SIRT1, which are known to promote mitochondrial biogenesis and support the body’s metabolic adaptation during stress conditions. AMPK activation enhances the expression of genes involved in fatty acid oxidation and mitochondrial biogenesis, while SIRT1, a NAD^+^-dependent deacetylase, increases mitochondrial function by deacetylating key proteins involved in energy metabolism, such as PGC-1α and SIRT3, thus optimizing oxidative metabolism and promoting energy efficiency [153].

In conclusion, THRs are integral to energy metabolism and thermogenesis, particularly through their regulation of UCP1 in BAT and enhancement of mitochondrial efficiency in muscle tissue. These processes are supported by key pathways like AMPK and SIRT1, which optimize the body’s ability to adapt to metabolic stress, contributing to improved energy balance, thermogenesis, and overall metabolic health. Thus, THRs are crucial in maintaining energy homeostasis and supporting endurance through efficient mitochondrial function.

#### Liver dysfunction, MASLD, and MASH

THRs play crucial roles in regulating lipid metabolism, fatty acid oxidation, and liver fibrosis, significantly impacting the pathophysiology of MASLD and MASH. THRα promotes fatty acid oxidation and lipid clearance in the liver by activating pathways like AMPK and PPARα, which enhance mitochondrial function and stimulate β-oxidation of fatty acids [154].

However, dysfunction in THRα signaling leads to lipid accumulation and steatosis due to impaired fatty acid oxidation, contributing to the progression of MASLD [155]. In contrast, THRβ, through its interaction with PPARα, regulates fatty acid oxidation and helps prevent lipotoxicity, a key factor in the development of MASH [156]. THRβ signaling activates PPARα, which modulates the expression of genes involved in fatty acid transport, mitochondrial biogenesis, and oxidation, ensuring efficient lipid processing and reducing the accumulation of toxic lipid intermediates in the liver. Impaired THRβ signaling exacerbates inflammation and fibrosis by promoting hepatic stellate cell activation, a crucial step in liver fibrosis. This occurs through the modulation of transforming growth factor beta (TGF-β) signaling, which stimulates the fibrogenic response in hepatocytes and surrounding tissues [157].

Recent studies also highlight that THRs enhance mitochondrial function and hepatic lipid metabolism by upregulating genes involved in the electron transport chain, such as NDUFA4 and CYC1, through SIRT1 [158]. This helps reduce hepatic fat accumulation and supports cellular energy production, particularly during periods of metabolic stress. Additionally, THRs modulate inflammatory pathways by suppressing pro-inflammatory cytokines like TNF-α, IL-6, and IL-1β via the NF-κB pathway. THRs influence the NF-κB pathway by interacting with the IKK complex, reducing the activation of NF-κB and the downstream expression of inflammatory cytokines, which are elevated in liver diseases like MASLD and MASH [159].

Furthermore, THRs interact with liver LXRs to regulate cholesterol and lipid homeostasis, coordinating the balance between lipid accumulation and efflux in hepatocytes [160]. Activation of LXRα by thyroid hormones promotes the expression of genes like ABCA1 and CYP7A1, which are involved in cholesterol efflux and bile acid synthesis, further supporting the reduction of hepatic fat and liver inflammation [161].

Targeting THR signaling, particularly through THRβ agonists, shows significant therapeutic potential for managing MASLD and MASH by enhancing fatty acid oxidation, reducing hepatic fat, and modulating inflammation. THRβ agonists have been shown to improve mitochondrial function, suppress NF-κB signaling, and promote lipid homeostasis, offering a promising strategy for treating metabolic liver diseases. Thus, THRs are valuable targets in the management of MASLD and MASH, as they help regulate lipid metabolism, reduce liver inflammation, and promote mitochondrial health.

#### Interaction with the gut–liver axis

THRs play a crucial role in the gut–liver axis, regulating lipid metabolism, bile acid synthesis, and inflammation, all of which are essential for liver function and metabolic health. THRβ influences bile acid synthesis by modulating receptors such as FXR and TGR5, ensuring proper bile acid levels [162]. Activation of FXR by thyroid hormones regulates the transcription of genes involved in bile acid synthesis and transport, such as CYP7A1 and bile salt export pump (BSEP), which collectively affect bile acid pools in the liver and intestine, influencing liver disease progression [163]. THRβ signaling impacts lipid metabolism and intestinal health by modulating bile acid homeostasis, which in turn shapes the gut microbiome composition, affecting gut microbiota diversity and influencing hepatic lipid metabolism.

In addition to regulating bile acid levels, THR signaling also modulates the production of SCFAs, such as butyrate, which are crucial for hepatic lipid metabolism, fatty acid oxidation, and insulin sensitivity. SCFAs activate the GPR41/43 signaling pathway and influence AMPK and PPARα pathways, promoting fatty acid oxidation and glucose homeostasis [164]. These metabolites, produced by gut microbiota, enhance the liver’s ability to process lipids efficiently, preventing fatty acid accumulation and contributing to metabolic health.

THRs also regulate gut-derived endotoxemia, which plays a significant role in liver inflammation and disease progression. By influencing intestinal permeability through the tight junction proteins (such as claudin-1 and occludin) and NF-κB pathways, THRs help prevent the entry of endotoxins like LPS into the bloodstream [165]. This reduction in endotoxin translocation suppresses systemic inflammation and protects against chronic liver conditions, including MASLD and MASH.

Additionally, THR signaling helps regulate pro-inflammatory cytokines such as TNF-α, IL-6, and IL-1β by modulating the NF-κB and c-Jun N-terminal kinase (JNK) pathways. Enhanced THR activity suppresses the expression of these cytokines, reducing liver inflammation and mitigating chronic liver damage [166].

Given these mechanisms, targeting THR signaling, particularly through THR agonists or modifying the gut microbiome, holds therapeutic potential for managing liver-related metabolic diseases. Restoring gut–liver homeostasis, reducing lipid accumulation, and limiting inflammation can offer effective strategies for treating conditions like MASLD, MASH, and other liver diseases influenced by thyroid hormone signaling.

#### Role in cancer progression

THRs are vital regulators of cell proliferation, differentiation, and apoptosis, and their dysregulation contributes to cancer initiation and progression. THRs function as transcription factors, binding to TREs in DNA to regulate genes involved in cell cycle control and survival pathways, such as cyclins, CDKs, and p53, through key signaling pathways like MAPK and PI3K/Akt [167]. Disrupted THR signaling leads to the overexpression of genes that promote cell proliferation and inhibit apoptosis, facilitating uncontrolled tumor growth. Additionally, THRs modulate angiogenesis by regulating the expression of VEGF, promoting tumor vascularization and metastasis [168]. THR signaling also influences matrix metalloproteinases (MMPs), particularly MMP-2 and MMP-9, through transcription factors like NF-κB and AP-1, which promote tumor invasion [169]. THRs further impact the tumor microenvironment by regulating cytokines such as TNF-α and IL-6, contributing to immune evasion and cancer progression [170]. By targeting THR pathways, these therapies aim to reduce tumor growth and improve treatment outcomes in various cancers, offering a promising strategy for cancer management.

#### Role in neuroprotection and neurodegenerative diseases

THRs are critical regulators of neuronal survival, synaptic plasticity, and neurotrophic factor signaling, contributing to brain function and offering protection against neurodegeneration. THR activation enhances the expression of brain-derived neurotrophic factor (BDNF) through MAPK/ERK signaling, promoting neuronal survival, growth, and repair, which is particularly important in neurodegenerative diseases such as AD and PD [171]. THR signaling also influences synaptic plasticity by modulating pathways like CREB, which is essential for memory and learning [172]. CREB activation, in turn, enhances the transcription of genes involved in long-term potentiation and long-term depression, both crucial for synaptic remodeling [173]. Additionally, THRs regulate antioxidant defenses by upregulating the expression of superoxide dismutase (SOD) and glutathione peroxidase, key enzymes that protect neurons from oxidative stress through the Nrf2 pathway [174]. By activating Nrf2, TRs promote the transcription of genes involved in oxidative stress response, preventing neuronal damage. THRs also modulate inflammatory pathways, reducing neuroinflammation by regulating pro-inflammatory cytokines such as TNF-α and IL-6 through the NF-κB and JNK signaling pathways, which are elevated in neurodegenerative conditions [175]. Furthermore, THRs enhance mitochondrial function by promoting the expression of genes involved in mitochondrial biogenesis and energy production, such as those regulated by the PGC-1α pathway, ensuring optimal neuronal metabolism. In AD and PD, THRs help regulate amyloid precursor protein (APP) processing via GSK-3β and reduce toxic Aβ plaque accumulation, while in PD, THRs support dopamine synthesis through the regulation of tyrosine hydroxylase [176]. By influencing these diverse pathways, THR signaling plays a vital neuroprotective role in enhancing neuronal repair, supporting mitochondrial function, and reducing neuroinflammation, offering significant therapeutic potential for treating neurodegenerative diseases.

#### Role in inflammation regulation

THRs play a significant role in modulating the inflammatory response by regulating the expression of pro-inflammatory cytokines such as TNF-α and IL-6, key mediators of inflammation. THRα and THRβ modulate various inflammatory pathways, including the NF-κB and AP-1 transcription factors, which control the production of these cytokines. Upon activation by thyroid hormones, THRs suppress NF-κB activation, reducing the expression of inflammatory mediators and cytokines. Specifically, THRα inhibits the IKK complex, preventing the activation of NF-κB and the subsequent production of pro-inflammatory cytokines [177]. In parallel, THRs influence AP-1 activity by modulating c-Jun and c-Fos, transcription factors that contribute to cytokine production. Additionally, THR signaling regulates the activity of immune cells such as macrophages and T cells, modulating their activation and cytokine secretion through pathways like MAPK and PI3K/Akt [178]. In conditions such as hypothyroidism or hyperthyroidism, dysregulation of THR activity can lead to an imbalance in inflammatory responses, contributing to excessive inflammation. This can exacerbate autoimmune disorders, CVDs, and other chronic inflammatory conditions. By controlling cytokine production and immune cell function, TRs are essential for maintaining immune homeostasis and preventing chronic inflammation.

#### Role in cardioprotection

THRs are critical in maintaining cardiovascular health by regulating heart rate, cardiac contractility, and vascular function. THR signaling modulates gene expression involved in myocardial energy metabolism, ensuring an adequate energy supply to the heart, especially under stress conditions like ischemia. THRs influence ATP production, fatty acid oxidation, and mitochondrial function through key signaling pathways, such as AMPK and PPARα, which are essential for sustaining the heart’s energy demands during periods of high workload or reduced oxygen supply [179]. THRα, in particular, regulates cardiac contractility and heart rate via the CaMKII pathway, which modulates the force of contraction and cardiac rhythm [180]. THRβ plays a significant role in controlling vascular function and blood pressure by influencing the expression of eNOS and NO production, which are critical for vascular relaxation and blood flow regulation [181]. THR agonists have shown cardioprotective effects through the activation of these pathways by enhancing cardiac function, improving myocardial metabolism, and reducing ischemia-induced damage. These agonists help preserve myocardial tissue by increasing the efficiency of energy utilization and mitigating the adverse effects of ischemic injury, partially via increased mitochondrial biogenesis through the SIRT1-mediated pathway. Furthermore, THR signaling improves vascular reactivity by promoting NO-mediated vasodilation, enhancing blood flow during stress, and preventing cardiovascular damage. Through these pathways, THRs contribute to cardioprotection, ensuring proper heart function and reducing ischemic damage.

#### Conclusion

THRα and THRβ are key regulators of metabolic, hepatic, cardiovascular, and neurological functions. They maintain glucose balance, enhance insulin sensitivity, and support mitochondrial function via AMPK, SIRT1, and PI3K/Akt pathways. In the liver, they control lipid metabolism, prevent MASLD/MASH, and regulate inflammation. THRs also influence gut–liver interactions, cancer progression, neuroprotection, immune modulation, and cardiovascular health. Their broad regulatory roles make them valuable therapeutic targets for metabolic, inflammatory, degenerative, and CVDs.

### Estrogen-related receptor alpha (ERRα)

#### Glucose homeostasis and insulin secretion

ERRα is a ligand-independent NR that orchestrates glucose homeostasis and insulin secretion through tissue-specific transcriptional regulation. Primarily interacting with coactivators PGC-1α and PGC-1β, ERRα activates genes involved in mitochondrial biogenesis, oxidative phosphorylation, and glucose metabolism. In the liver, ERRα promotes gluconeogenesis by upregulating PEPCK and G6Pase via the PGC-1α–FOXO1 and CREB/cAMP pathways [182].

In skeletal muscle, it enhances GLUT4 expression and glucose utilization through the PI3K/Akt and AMPK pathways, while also supporting mitochondrial efficiency via NRF1/NRF2 [183]. In pancreatic β-cells, ERRα boosts insulin secretion by increasing mitochondrial ATP production and repressing UCP2, thereby sustaining the ATP/ADP ratio essential for insulin granule exocytosis [184]. Collectively, ERRα integrates metabolic signals to regulate glucose output, uptake, and insulin action, positioning it as a promising therapeutic target for insulin resistance, T2DM, and metabolic syndrome.

#### Liver dysfunction, MASLD, and MASH

ERRα is a pivotal regulator of hepatic energy metabolism, coordinating mitochondrial function, fatty acid oxidation, inflammation, and fibrosis. In the liver, ERRα, primarily through its interaction with PGC-1α and activation of the AMPK/SIRT1 axis, enhances expression of genes involved in β-oxidation and oxidative phosphorylation, thereby preventing triglyceride accumulation and mitochondrial dysfunction typical of early MASLD. It also suppresses NF-κB-mediated inflammatory signaling and promotes antioxidant defense, limiting cytokine production and oxidative stress that drive hepatocyte injury and MASH progression [185].

Additionally, ERRα modulates fibrosis by inhibiting TGF-β/Smad3 signaling and controls cholesterol and bile acid homeostasis via CYP7A1 and LXR interaction [186]. Crosstalk with NRs like PPARα, FXR, and HNF4α enables ERRα to adapt hepatic gene expression to metabolic stress. While ERRα activation is protective in early disease stages, its downregulation in advanced MASH exacerbates mitochondrial dysfunction, inflammation, and fibrosis. Thus, ERRα is a promising therapeutic target for MASLD/MASH, though careful modulation is necessary to avoid mitochondrial overload.

#### Interaction with gut–liver axis

ERRα is a key transcriptional regulator of the gut–liver axis, integrating metabolic, microbial, and inflammatory signals to maintain systemic homeostasis. In the liver, ERRα—via coactivation with PGC-1α—regulates bile acid synthesis genes like CYP7A1 and CYP8B1, indirectly modulating FXR/TGR5 signaling and shaping gut microbiota composition [156]. These microbial shifts influence SCFA production, which in turn activates the AMPK–SIRT1–PGC-1α axis, enhancing ERRα-driven mitochondrial function and gene transcription in both liver and intestinal epithelium. ERRα supports gut barrier integrity by promoting tight junction proteins and antioxidant defenses, preventing endotoxin translocation that triggers hepatic inflammation. It also buffers hepatic responses to microbial metabolites by enhancing fatty acid oxidation and suppressing NF-κB-mediated inflammation [187]. Thus, ERRα serves as a metabolic integrator between gut-derived cues and liver physiology, and its therapeutic targeting—through agonists, prebiotics, or metabolic interventions—holds promise for treating MASLD, MASH, and related metabolic disorders.

#### Role in cancer progression

ERRα plays a pivotal role in cancer progression by acting as a central metabolic and transcriptional regulator that enables tumor cells to adapt to environmental stress and sustain malignancy. In cooperation with its coactivator PGC-1α, ERRα promotes the transcription of metabolic genes that support cancer cell energy demands, including HK2 and LDHA for glycolysis, CPT1A for fatty acid oxidation, and COX4I1 and ATP5B for mitochondrial oxidative phosphorylation. This metabolic reprogramming, largely mediated through the AMPK–PGC-1α–ERRα axis, fuels rapid proliferation under nutrient-limited or hypoxic conditions [188].

Beyond metabolism, ERRα enhances tumor cell survival by upregulating anti-apoptotic and proliferative genes such as BCL2, Cyclin D1, and c-Myc, and by activating key oncogenic signaling pathways like PI3K/Akt/mTOR and MAPK/ERK. In hypoxic regions of tumors, ERRα cooperates with HIF-1α to stimulate angiogenesis via induction of angiogenic factors such as VEGF-A and ANGPTL4, ensuring adequate oxygen and nutrient supply [189].

Furthermore, ERRα promotes EMT and metastatic potential by regulating genes involved in cell motility and extracellular matrix remodeling, including MMP2, MMP9, Snail, and Twist, through the NF-κB, TGF-β/Smad, and FAK/Src pathways. It also contributes to immune evasion and chronic inflammation by modulating cytokine expression, particularly IL-6 and TNF-α, thereby creating a tumor-promoting microenvironment [190].

Critically, ERRα is implicated in therapy resistance by maintaining mitochondrial redox balance, enhancing DNA repair mechanisms, and promoting metabolic flexibility that enables tumor cells to survive oxidative stress induced by chemotherapy or radiation. Elevated ERRα expression is consistently associated with poor prognosis and treatment resistance across various cancers, highlighting its potential as a therapeutic target in oncology.

#### Role in neuroprotection and neurodegenerative diseases

ERRα plays a critical neuroprotective role by regulating energy metabolism, oxidative stress, synaptic integrity, inflammation, and neuronal survival—core processes often impaired in neurodegenerative diseases such as AD, PD, and Huntington’s disease (HD). In coordination with its coactivator PGC-1α, ERRα promotes mitochondrial biogenesis and oxidative phosphorylation by upregulating genes such as ATP5B, COX4, NDUFA9, and CYC1, thereby sustaining ATP production to meet neuronal and synaptic energy demands. This mitochondrial support is reinforced through the AMPK–SIRT1–PGC-1α–ERRα signaling axis, which is especially vital during neuronal energy stress and early mitochondrial dysfunction characteristic of AD and PD [191].

ERRα also counters oxidative stress by enhancing expression of key antioxidant enzymes, including SOD2, GPx1, and Catalase, and may cooperate with Nrf2 to strengthen cellular redox defenses. At the synaptic level, ERRα promotes plasticity and cognitive function by regulating BDNF, Synaptophysin, and PSD-95, in part via the CREB pathway, while potentially influencing cholinergic and dopaminergic signaling [192]. To limit neuroinflammation, ERRα suppresses NF-κB-mediated transcription of pro-inflammatory cytokines such as TNF-α, IL-1β, and IL-6, and may modulate microglial polarization toward an anti-inflammatory phenotype, with additional contributions from PPARγ and SIRT1 [193].

In preventing neuronal apoptosis, ERRα upregulates survival genes like BCL2 and Survivin, while downregulating pro-apoptotic factors such as BAX and Caspase-3, maintaining mitochondrial membrane integrity through PI3K/Akt and SIRT3 pathways. Experimental studies reinforce these roles: ERRα deficiency leads to impaired cognition and mitochondrial dysfunction, whereas its activation improves mitochondrial efficiency, reduces oxidative damage, and enhances behavioral outcomes [194]. Collectively, these actions establish ERRα as a compelling therapeutic target in the management of neurodegenerative diseases.

#### Role in cardioprotection

ERRα plays a vital cardioprotective role by regulating cardiac energy metabolism, mitochondrial function, oxidative stress response, and vascular homeostasis. As a ligand-independent NR, ERRα is highly expressed in the heart, where it orchestrates transcriptional programs essential for meeting the high energy demands of cardiac tissue. Its cardioprotective effects are primarily mediated through its interaction with the coactivator PGC-1α, forming the PGC-1α–ERRα axis, which governs mitochondrial biogenesis and oxidative phosphorylation. This axis drives the expression of key mitochondrial genes including ATP5A1, COX4, NDUFA9, and CYC1, enhancing ATP production and supporting contractile function, especially during stress conditions such as ischemia or increased workload [195].

In energy-stressed myocardium, ERRα integrates upstream signals from AMPK and SIRT1, which activate PGC-1α and enhance ERRα-mediated transcription. This signaling loop ensures mitochondrial efficiency, fatty acid oxidation, and antioxidant capacity are maintained, reducing ischemia-induced damage. ERRα also upregulates enzymes like CPT1B and MCAD, facilitating fatty acid β-oxidation—the heart's primary energy source—thereby preserving cardiac function under metabolic stress [196].

Beyond metabolism, ERRα contributes to redox homeostasis by inducing antioxidant enzymes such as SOD2 and GPx1, limiting reactive oxygen species (ROS) accumulation and oxidative injury. Additionally, ERRα modulates vascular function by eNOS expression and NO production, supporting vasodilation and blood pressure regulation [197].

ERRα further inhibits cardiac inflammation by downregulating pro-inflammatory cytokines such as TNF-α and IL-6 through NF-κB suppression, thereby protecting against inflammatory damage associated with heart failure and atherosclerosis. In models of ischemic injury, ERRα activation enhances myocardial recovery by preserving mitochondrial structure, improving oxidative metabolism, and reducing apoptosis through pathways involving PI3K/Akt and SIRT3 [198].

In summary, ERRα confers cardioprotection by acting as a metabolic and transcriptional hub that maintains mitochondrial function, energy supply, redox balance, and vascular integrity. Through the integrated action of the AMPK–SIRT1–PGC-1α–ERRα axis and its anti-inflammatory and anti-apoptotic effects, ERRα helps safeguard cardiac tissue against stress-induced dysfunction and damage, making it a promising therapeutic target in CVD.

#### Role in inflammation regulation

ERRα is a critical immunometabolic regulator that integrates inflammatory signaling with cellular energy status, playing a central role in maintaining immune homeostasis, particularly in metabolically active tissues such as the liver, adipose tissue, and brain. Although primarily recognized for its metabolic functions, ERRα also suppresses pathological inflammation through transcriptional and signaling mechanisms. At the transcriptional level, ERRα downregulates key pro-inflammatory cytokines—TNF-α, IL-6, IL-1β, and MCP-1—by modulating or competing with transcription factors such as NF-κB and AP-1. ERRα represses inflammatory gene expression either by directly interfering with these transcriptional complexes or by limiting their access to coactivators [199].

A central mechanism of ERRα’s anti-inflammatory action involves its inhibition of the NF-κB pathway. ERRα suppresses IKKβ activity, preventing the degradation of IκB, thereby retaining the p65/RelA subunit in the cytoplasm and blocking NF-κB-driven transcription of inflammatory mediators like TNF-α and COX-2. This anti-inflammatory action is further enhanced through crosstalk with metabolic regulators. The AMPK–SIRT1–PGC-1α–ERRα axis links energy sensing with immune modulation: AMPK activates ERRα indirectly via PGC-1α phosphorylation, while SIRT1 deacetylates both PGC-1α and NF-κB, attenuating inflammatory responses and supporting mitochondrial function essential for resolving immune stress [200].

ERRα also modulates immune cell behavior. In macrophages, it promotes polarization toward the anti-inflammatory M2 phenotype and suppresses M1-associated cytokine production and ROS generation. In the central nervous system, ERRα reduces microglial activation and cytokine secretion, contributing to neuroimmune stability. Although less explored, ERRα may also influence T-cell differentiation and function under metabolic or oxidative stress conditions [201].

These actions manifest in tissue-specific contexts. In adipose tissue, ERRα reduces obesity-induced inflammation and improves insulin signaling. In the liver, it inhibits NF-κB-mediated cytokine production, limiting hepatic injury and Kupffer cell activation in MASLD and MASH. In the brain, ERRα restrains glial inflammation and supports neuronal integrity during neurodegenerative processes. Experimental models reinforce these findings: ERRα knockout animals show heightened inflammatory responses, while ERRα agonists reduce cytokine expression and inflammation in models of obesity, atherosclerosis, and neuroinflammation [202].

In summary, ERRα acts as a pivotal transcriptional and metabolic regulator of inflammation. By suppressing inflammatory transcription factors, interacting with AMPK/SIRT1 metabolic pathways, and modulating immune cell function, ERRα protects against chronic inflammation in diverse pathological states. Its strategic positioning at the intersection of metabolism and immunity makes it a promising target for treating inflammation-driven disorders.

#### Role in mitochondrial function and oxidative metabolism

ERRα plays a crucial role in mitochondrial function and oxidative metabolism. Unlike classical ERs, ERRα is regulated by coactivators and PTMs, rather than direct ligand activation. Highly expressed in skeletal muscle, heart, and brown adipose tissue (BAT), ERRα promotes mitochondrial biogenesis, fatty acid oxidation, and adaptive thermogenesis. It regulates genes involved in mitochondrial respiration, including cytochrome c oxidase and ATP synthase. Furthermore, ERRα controls UCP1 and PGC-1α expression in BAT, linking its activity to thermogenic processes and energy expenditure [203].

As an emerging therapeutic target for metabolic diseases and age-related metabolic decline, ERRα holds promise for treating conditions such as obesity, muscle wasting, and CVDs, by improving mitochondrial function and oxidative metabolism.

#### Conclusion

ERRα is a key transcriptional regulator linking metabolic and inflammatory signaling across tissues. Through coactivators like PGC-1α and pathways such as AMPK and SIRT1, it promotes mitochondrial biogenesis, oxidative phosphorylation, fatty acid oxidation, thermogenesis, and supports glucose and lipid metabolism, insulin sensitivity, and NF-κB–mediated anti-inflammatory effects. In conditions including diabetes, MASLD/MASH, CVD, cancer, and neurodegeneration, ERRα helps restore energy balance, reduce oxidative and inflammatory stress, and preserve tissue function, making it a promising therapeutic target for metabolic, inflammatory, and age-related diseases.

### PGC-1α (peroxisome proliferator-activated receptor gamma coactivator 1 alpha)

#### Glucose homeostasis and insulin secretion

PGC-1α is a key transcriptional coactivator that regulates genes involved in energy metabolism, mitochondrial function, and cellular stress responses. In the context of glucose homeostasis and insulin secretion, PGC-1α plays a critical role by integrating various metabolic pathways that control glucose production, insulin sensitivity, and beta-cell function in the pancreas. In the liver, PGC-1α promotes gluconeogenesis by activating key enzymes such as PEPCK and G6Pase, which are essential for glucose production during fasting. It achieves this through interactions with transcription factors like FOXO1 and CREB, enhancing the expression of gluconeogenic genes in response to energy demand. This regulation is crucial for maintaining blood glucose levels during periods of fasting or exercise [204].

In skeletal muscle, PGC-1α enhances insulin sensitivity by promoting the expression of GLUT4, facilitating glucose uptake into muscle cells in response to insulin. Additionally, PGC-1α helps optimize mitochondrial function and fatty acid oxidation, contributing to better glucose utilization and metabolic flexibility. This is particularly important for maintaining glucose homeostasis, as muscle tissue accounts for a large portion of the body's glucose uptake [204].

In the pancreatic β-cells, PGC-1α plays a pivotal role in regulating insulin secretion. It modulates mitochondrial function and ATP production, which is crucial for insulin granule exocytosis. By enhancing mitochondrial biogenesis and improving ATP/ADP ratio, PGC-1α supports insulin release in response to glucose stimulation. Moreover, PGC-1α regulates the expression of genes involved in mitochondrial oxidative phosphorylation, ensuring that β-cells have sufficient energy for insulin production and secretion [205].

PGC-1α also influences insulin sensitivity systemically by improving the function of IRS-1 and IRS-2 and activating AMPK, a key regulator of cellular energy balance. Through these pathways, PGC-1α enhances glucose uptake and regulates insulin action, ensuring proper glucose utilization and preventing insulin resistance [206].

In summary, PGC-1α integrates multiple metabolic pathways to regulate glucose homeostasis and insulin secretion. In the liver, it drives gluconeogenesis, in skeletal muscle, it enhances insulin sensitivity and glucose utilization, and in pancreatic β-cells, it supports insulin secretion by promoting mitochondrial function and ATP production. These actions position PGC-1α as a critical regulator of metabolic health, with potential therapeutic applications for insulin resistance, T2DM, and metabolic syndrome.

#### Liver dysfunction, MASLD, and MASH

PGC-1α is a key regulator of mitochondrial function and energy metabolism, playing a central role in the liver’s response to metabolic stress and liver dysfunction. In conditions such as MASLD and MASH, PGC-1α helps maintain mitochondrial integrity, oxidative metabolism, and cellular homeostasis, which are compromised in these diseases.

In MASLD, where excessive lipid accumulation in hepatocytes occurs, PGC-1α regulates key genes involved in fatty acid oxidation and mitochondrial biogenesis. By enhancing the expression of CPT1A and acyl-CoA dehydrogenases, PGC-1α facilitates fatty acid β-oxidation, which helps prevent lipid accumulation and the onset of steatosis [207]. PGC-1α also stimulates the transcription of genes involved in mitochondrial oxidative phosphorylation, ensuring that the liver efficiently utilizes fatty acids for energy production. This is crucial in preventing hepatic lipid overload and the subsequent progression to MASH.

In MASH, characterized by inflammation, oxidative stress, and liver fibrosis, PGC-1α acts as a regulator of inflammation and oxidative stress. PGC-1α promotes the expression of antioxidant genes, such as SOD2 and GPx1, which help mitigate ROS accumulation. By reducing ROS-induced damage, PGC-1α helps protect hepatocytes from apoptosis and prevents the activation of HSCs, which are key mediators of liver fibrosis. Additionally, PGC-1α modulates NF-κB signaling, a central pathway in the inflammatory response, by inhibiting the production of pro-inflammatory cytokines like TNF-α and IL-6, which are elevated in MASH [208].

PGC-1α’s role in mitochondrial function is critical in both MaSLD and MaSH. By enhancing mitochondrial biogenesis through the activation of nuclear respiratory factor 1 (NRF1) and mitochondrial transcription factor A (TFAM), PGC-1α helps maintain mitochondrial number and function, crucial for energy production and cellular integrity. This process is particularly important in combating the metabolic dysfunction seen in liver disease [209].

Furthermore, PGC-1α regulates cholesterol and bile acid metabolism in the Liver, where it interacts with LXR and FXR, influencing cholesterol 7α-hydroxylase (CYP7A1) expression and bile acid synthesis. This regulation helps restore lipid homeostasis and reduce hepatic fat accumulation, which is often dysregulated in MASH [59].

In summary, PGC-1α exerts a protective effect in liver dysfunction, particularly in MASLD and MASH, by enhancing fatty acid oxidation, mitochondrial biogenesis, and antioxidant defenses, while simultaneously reducing inflammation and oxidative stress. These actions help prevent lipid accumulation, mitigate cellular damage, and inhibit fibrosis progression. Given these effects, PGC-1α represents a promising therapeutic target for managing MASLD, MASH, and other related liver disorders.

#### Interaction with the gut–liver axis

PGC-1α plays a pivotal role in maintaining metabolic homeostasis, and its influence extends beyond the liver to the gut–liver axis, a critical communication pathway between the gastrointestinal tract and the liver. This axis integrates signals from dietary nutrients, gut microbiota, inflammatory responses, and metabolic cues, and PGC-1α mediates many of these interactions to regulate energy balance, bile acid metabolism, and inflammatory responses.

At the liver, PGC-1α directly modulates bile acid synthesis by regulating CYP7A1, the rate-limiting enzyme in bile acid biosynthesis. Through this regulation, PGC-1α influences bile acid homeostasis, which is essential for the absorption of dietary lipids and fat-soluble vitamins in the intestine. PGC-1α’s activation in the liver also regulates genes involved in glucose and lipid metabolism, helping the liver process and store nutrients that enter from the gut, thus ensuring systemic energy balance [210].

Gut microbiota are deeply intertwined with PGC-1α's activity. By influencing the synthesis and secretion of bile acids, PGC-1α indirectly shapes the composition and activity of gut microbiota. Bile acids, in turn, serve as signaling molecules that regulate gut microbial diversity and composition by activating FXR and TGR5 receptors in the intestines. These interactions between bile acids and the gut microbiome help maintain intestinal health and modulate inflammatory responses in the gut, which can have a downstream impact on liver metabolism [211].

Furthermore, PGC-1α is involved in gut barrier function by modulating the expression of tight junction proteins like ZO-1 and occludin, which control intestinal permeability. By maintaining the integrity of the gut barrier, PGC-1α prevents the translocation of harmful microbial products, such as LPS, into the bloodstream, thus reducing systemic inflammation and protecting the liver from endotoxemia. The NF-κB pathway, which is activated by microbial products like LPS, is modulated by PGC-1α’s antioxidant functions and its regulation of mitochondrial biogenesis, helping resolve inflammation and protect both gut and liver cells [211]

The interaction of PGC-1α with the AMPK–SIRT1 axis is also critical in maintaining metabolic stability across the gut–liver axis. In response to nutrient availability or energy stress, AMPK activation in the liver and intestines enhances PGC-1α’s activity, promoting fatty acid oxidation, mitochondrial biogenesis, and glucose homeostasis. This not only supports the liver's ability to process nutrients but also enables the gut to adapt to fluctuations in energy supply, optimizing nutrient absorption and preventing metabolic dysregulation [212].

In summary, PGC-1α regulates the gut–liver axis through its effects on bile acid metabolism, mitochondrial function, and inflammatory responses. By modulating bile acid synthesis in the liver, PGC-1α influences gut microbial composition and bile acid signaling. In the gut, it preserves barrier integrity and reduces inflammation, protecting both the gut and liver from metabolic stress and endotoxemia. Through its coordination of metabolic pathways in both the liver and the intestine, PGC-1α helps integrate signals from the gut microbiome and dietary inputs, promoting systemic metabolic balance and offering a potential therapeutic target for metabolic diseases such as MASLD, MASH, and insulin resistance.

#### Role in cancer progression

PGC-1α plays a complex role in cancer progression by regulating cellular metabolism, mitochondrial function, and stress responses. Depending on the cancer type and microenvironment, PGC-1α can either suppress tumors or promote tumor survival and growth.

A key mechanism involves its regulation of mitochondrial function. PGC-1α drives mitochondrial biogenesis by activating genes involved in oxidative phosphorylation, such as COX4 and ATP5B, and by promoting fatty acid oxidation. In cancers characterized by the Warburg effect, PGC-1α can shift metabolism toward oxidative phosphorylation, increasing oxidative stress and ROS levels. This can induce DNA damage and trigger cell death pathways, potentially suppressing tumor growth [213].

PGC-1α also helps cancer cells adapt to stress. Through interactions with AMPK and SIRT1, PGC-1α coordinates cellular responses to nutrient availability and stress. AMPK activation under low energy conditions increases PGC-1α activity, enhancing mitochondrial biogenesis and metabolic flexibility. These adaptations enable cancer cells to survive in hypoxic and nutrient-deprived environments, supporting tumor progression and metastasis [214].

Additionally, PGC-1α regulates tumor suppressor pathways, including those mediated by p53. In response to DNA damage, PGC-1α facilitates p53-mediated apoptosis by regulating genes involved in mitochondrial stress responses. However, in some cancers, PGC-1α downregulation leads to mitochondrial dysfunction and reduced apoptosis, allowing cancer cells to evade cell death and resist chemotherapy [215].

PGC-1α also interacts with chromatin remodeling complexes and HDACs, influencing epigenetic regulation and gene expression. By enhancing tumor suppressor gene expression and repressing oncogenes, PGC-1α controls cancer cell growth, invasion, and metastasis [216].

Furthermore, PGC-1α modulates inflammatory signaling pathways, such as NF-κB, which plays a key role in inflammation-associated cancer progression. By regulating NF-κB signaling, PGC-1α can reduce pro-inflammatory cytokine production, potentially suppressing inflammation-driven tumor progression and metastasis [217].

In summary, PGC-1α acts as both a tumor suppressor and promoter, depending on context. It can enhance mitochondrial function, promote apoptosis, and regulate metabolic pathways to limit cancer cell survival, while also supporting tumor growth by promoting metabolic flexibility and stress adaptation. By influencing mitochondrial biogenesis, fatty acid oxidation, tumor suppressor pathways, and epigenetic regulation, PGC-1α serves as a central regulator of cancer cell metabolism and is a promising therapeutic target for cancer progression and chemoresistance.

#### Role in neuroprotection and neurodegenerative diseases

PGC-1α is a crucial transcriptional coactivator that regulates mitochondrial biogenesis, energy metabolism, and cellular stress responses. It plays a vital role in neuroprotection and is implicated in neurodegenerative diseases such as AD, PD, and HD by promoting mitochondrial function, enhancing antioxidant defenses, modulating inflammation, and supporting neuronal survival.

A primary neuroprotective role of PGC-1α is its regulation of mitochondrial biogenesis and function. Mitochondrial dysfunction is a hallmark of neurodegenerative diseases, contributing to neuronal death. PGC-1α drives the expression of mitochondrial genes involved in oxidative phosphorylation, including COX4, ATP5B, and NDUFA9, ensuring efficient ATP production. By promoting mitochondrial biogenesis and regulating mitochondrial dynamics, PGC-1α increases neuronal energy supply and maintains mitochondrial integrity, helping prevent excessive oxidative damage and neurodegeneration [218].

PGC-1α also plays a critical role in managing oxidative stress, which contributes to neuronal damage in neurodegenerative diseases. It activates antioxidant genes such as SOD2, GPx1, and Catalase, which detoxify ROS and maintain redox homeostasis. By reducing oxidative damage, PGC-1α protects neurons in conditions like AD and PD [219].

In addition, PGC-1α helps control neuroinflammation, a central feature of neurodegeneration. Chronic activation of microglia and astrocytes leads to the release of pro-inflammatory cytokines like TNF-α, IL-1β, and IL-6, which exacerbate neuronal injury. PGC-1α inhibits NF-κB signaling, reducing the expression of these cytokines and mitigating neuroinflammation. It also promotes anti-inflammatory microglial polarization (M2 phenotype), further protecting neurons [220].

PGC-1α supports neuronal survival by regulating cell cycle checkpoints and apoptotic pathways. In neurodegenerative diseases, failure of these mechanisms contributes to neuronal loss. PGC-1α upregulates anti-apoptotic proteins, including BCL2 and Survivin, while downregulating pro-apoptotic factors like BAX and Caspase-3, thereby protecting neurons under stress conditions [221].

Finally, PGC-1α influences synaptic plasticity and neurotrophic support. It enhances the expression of BDNF, which is essential for synaptic maintenance and neurogenesis. Reduced BDNF levels are associated with cognitive decline in neurodegenerative diseases, and by regulating BDNF, PGC-1α helps preserve synaptic function and cognitive health [191].

In summary, PGC-1α protects neurons by promoting mitochondrial health, reducing oxidative stress and inflammation, supporting survival pathways, and enhancing synaptic plasticity. These actions make PGC-1α a key player in preventing neurodegeneration and a promising therapeutic target for slowing the progression of AD, PD, and HD.

#### Role in inflammation regulation

PGC-1α plays a crucial role in regulating inflammation, especially in metabolically active tissues such as the liver, adipose tissue, and skeletal muscle. While primarily known for its role in mitochondrial biogenesis and energy metabolism, PGC-1α also exerts anti-inflammatory effects that are essential for maintaining cellular homeostasis and preventing chronic inflammation linked to metabolic diseases, autoimmune conditions, and CVDs.

A key mechanism of PGC-1α’s anti-inflammatory action is its suppression of the NF-κB pathway, a central driver of inflammation. NF-κB regulates the transcription of pro-inflammatory cytokines, including TNF-α, IL-1β, and IL-6, which are elevated in chronic inflammatory states. PGC-1α inhibits NF-κB signaling by interacting with CoREST and recruiting HDACs and LSD1 to the promoters of NF-κB target genes, thereby preventing the transcription of these cytokines and limiting prolonged inflammatory responses [222].

PGC-1α also reduces inflammation by managing oxidative stress, which often triggers inflammatory pathways under metabolic stress. By upregulating antioxidant genes such as SOD2 and GPx1, PGC-1α detoxifies ROS and restores redox balance, preventing ROS-induced inflammation and cellular damage [223].

Additionally, PGC-1α modulates immune cell function, particularly in macrophages. It promotes M2 anti-inflammatory macrophage polarization, which supports tissue repair and immune resolution, while reducing the M1 pro-inflammatory phenotype associated with chronic inflammation and tissue damage. This modulation helps lower pro-inflammatory cytokine production and supports tissue healing [224].

PGC-1α also integrates metabolic signals with inflammation regulation through its interactions with AMPK and SIRT1. AMPK activation enhances PGC-1α activity, increasing mitochondrial biogenesis and energy production, which facilitates inflammation resolution. SIRT1 deacetylates both PGC-1α and NF-κB, further attenuating inflammatory signaling. This crosstalk between PGC-1α, AMPK, and SIRT1 ensures a balanced immune response, preventing chronic inflammation while maintaining appropriate immune activation [225].

In summary, PGC-1α regulates inflammation by suppressing NF-κB activity, reducing oxidative stress, promoting anti-inflammatory immune responses, and integrating metabolic regulation with immune control. These functions position PGC-1α as a potential therapeutic target for managing chronic inflammation in diseases such as obesity, atherosclerosis, diabetes, and autoimmune conditions.

#### Role in cardioprotection

PGC-1α plays a crucial role in maintaining cardiac health by regulating mitochondrial biogenesis, energy metabolism, and cellular stress responses. Given the heart's high energy demands, particularly under stress conditions such as ischemia, PGC-1α ensures efficient ATP production, supports mitochondrial function, and protects cardiac cells from damage, making it a key factor in cardioprotection.

A primary mechanism by which PGC-1α protects the heart is through promoting mitochondrial biogenesis and oxidative phosphorylation. By activating mitochondrial genes such as ATP5A1, COX4, and NDUFA9, PGC-1α ensures that cardiac mitochondria produce sufficient ATP to sustain continuous contractile activity. This mitochondrial support is particularly important during ischemia, where energy depletion and mitochondrial dysfunction can lead to cell death. By enhancing fatty acid oxidation and mitochondrial efficiency, PGC-1α provides a stable energy supply, reducing the risk of ischemic injury [226].

PGC-1α also contributes to cardioprotection by maintaining redox balance within cardiac cells. Mitochondrial metabolism generates ROS, which can cause oxidative stress and damage cellular components if not controlled. PGC-1α increases the expression of antioxidant enzymes, including SOD2 and GPx1, which detoxify ROS and reduce oxidative damage. By preserving redox homeostasis, PGC-1α helps prevent oxidative stress from impairing cardiac function during stress conditions [223].

In addition, PGC-1α plays a role in regulating cardiac inflammation. Cardiovascular events such as myocardial infarction and heart failure often involve the activation of NF-κB signaling, leading to the production of pro-inflammatory cytokines that exacerbate tissue injury. PGC-1α inhibits NF-κB activation, reducing the expression of cytokines like TNF-α, IL-6, and IL-1β. By limiting inflammation, PGC-1α helps protect cardiac tissue, supports repair, and improves overall heart function following injury [227].

PGC-1α further protects the heart by modulating cell survival pathways. It promotes the expression of anti-apoptotic proteins such as BCL2 and Survivin while suppressing pro-apoptotic factors like BAX and Caspase-3, thereby preserving myocardial cells under stress conditions such as ischemia and oxidative injury. Additionally, PGC-1α enhances endothelial function by regulating eNOS and promoting NO production, which improves vascular relaxation, blood flow, and overall cardiovascular health [228].

PGC-1α also interacts with AMPK and SIRT1, two key regulators of cellular energy metabolism and stress responses. AMPK activation enhances PGC-1α activity, promoting fatty acid oxidation and mitochondrial biogenesis to support energy balance. SIRT1 deacetylates and activates PGC-1α in response to metabolic stress, allowing for fine-tuned regulation of mitochondrial function and energy metabolism. These interactions enable PGC-1α to adapt cardiac metabolism to changing energy demands, ensuring optimal heart function during stress [229].

In summary, PGC-1α is essential for cardioprotection through its regulation of mitochondrial biogenesis, oxidative stress reduction, inflammation suppression, and cell survival pathways. By enhancing mitochondrial function, maintaining redox balance, and reducing inflammation, PGC-1α supports cardiac tissue integrity under stress and injury. These roles highlight PGC-1α as a promising therapeutic target for improving heart function in conditions such as heart failure, myocardial infarction, and other cardiovascular diseases.

#### Role in mitochondrial biogenesis and metabolic adaptation

PGC-1α is a pivotal regulator of mitochondrial biogenesis and oxidative capacity, crucial for metabolic adaptation. By coactivating various NRs, including PPARs, THRs, ERRα, and NRF1/NRF2, PGC-1α governs fatty acid oxidation, mitochondrial function, and glucose metabolism. This gene coactivator is particularly important for endurance adaptation, helping cells adapt to increased energy demands during exercise. PGC-1α is also regulated by nutrient-sensing pathways such as AMPK, SIRT1, and mTOR, which link cellular energy status to mitochondrial function and metabolic regulation [230].

PGC-1α has garnered attention as a potential therapeutic target for treating metabolic diseases Such as type 2 diabetes, neurodegenerative disorders, and aging-associated metabolic decline. Modulators of PGC-1α are being explored for their ability to enhance mitochondrial function and energy metabolism, offering a novel approach to combating diseases related to metabolic dysfunction.

#### Conclusion

PGC-1α is a critical regulator of mitochondrial biogenesis, oxidative metabolism, and energy homeostasis. It integrates various metabolic pathways, such as fatty acid oxidation, glucose metabolism, and mitochondrial function, through its interaction with key NRs (e.g., PPARs, THRs, ERRα, NRF1/NRF2) and nutrient-sensing pathways like AMPK, SIRT1, and mTOR. PGC-1α plays a pivotal role in enhancing endurance adaptation, managing cellular stress, and regulating glucose and lipid metabolism, making it vital for metabolic health. Its functions extend to critical areas such as cardiovascular protection, neuroprotection, and inflammation regulation, where it helps mitigate oxidative stress, improve mitochondrial efficiency, and suppress harmful inflammation. The therapeutic potential of PGC-1α in treating metabolic disorders like T2DM, neurodegenerative diseases, and aging-related decline highlights its importance as a target for therapeutic interventions aimed at improving mitochondrial function and overall metabolic health (Table 3).

**Table 3:** Unveiling the roles of THR, ERRα, and PGC-1α receptors: critical regulators in metabolism, inflammation, and disease

Feature		THR		ERRα		PGC-1α
Primary expression sites	Liver, muscle, adipose tissue, pancreas		Skeletal muscle, heart, brown adipose tissue (BAT), liver		Liver, skeletal muscle, heart, brain	
Main functions	Regulates glucose production, insulin sensitivity, thermogenesis, mitochondrial function		Regulates glucose metabolism, mitochondrial function, energy metabolism		Regulates mitochondrial biogenesis, glucose and lipid metabolism, cellular stress responses	
Activation mechanisms	Activated by thyroid hormones (T3, T4), THR agonists		Ligand-independent, activated by coactivators (e.g., PGC-1α)		Activated by nutrient-sensing pathways (AMPK, SIRT1, mTOR), PGC-1Î ± coactivators	
Target genes/proteins	PEPCK, G6Pase, GLUT4, IRS1/2		PEPCK, G6Pase, GLUT4, HK2, LDHA, CPT1A, COX4I1		PEPCK, G6Pase, GLUT4, PPARα, CPT1A, SOD2, GPx1, BDNF	
Metabolic role	Regulates glucose homeostasis, enhances insulin sensitivity, thermogenesis		Enhances glucose uptake, supports mitochondrial biogenesis, regulates fatty acid oxidation		Enhances mitochondrial function, regulates glucose and fatty acid metabolism, supports energy balance	
Anti-inflammatory role	Regulates cytokine production, suppresses NF-kB signaling		Modulates inflammatory pathways, suppresses pro-inflammatory cytokines		Suppresses inflammation through NF-kB inhibition, modulates immune cell function	
Therapeutic applications	THR agonists for diabetes, metabolic syndrome, NAFLD/NASH		ERRα agonists for insulin resistance, metabolic diseases, cancer therapy		PGC-1α activators for diabetes, NAFLD/NASH, neurodegenerative diseases, aging	
Diseases/conditions targeted	Hypothyroidism, hyperthyroidism, diabetes, metabolic syndrome, liver diseases		Cancer, diabetes, cardiovascular disease, neurodegenerative diseases		Type 2 diabetes, NAFLD, NASH, neurodegenerative diseases, cardiovascular diseases	

*THR* Thyroid hormone receptors, *ERRα* estrogen-related receptor alpha, *PGC-1α* peroxisome proliferator-activated receptor gamma coactivator 1 alpha, *PEPCK* phosphoenolpyruvate carboxykinase, *G6Pase* glucose-6-phosphatase, *GLUT4* glucose transporter type 4, *IRS1/2* insulin receptor Substrate 1/2, *AMPK* AMP-activated protein kinase, *mTOR* mechanistic target of rapamycin, *HK2* Hexokinase 2, *LDHA* lactate dehydrogenase A, *CPT1A* carnitine palmitoyltransferase 1A, *COX4I1* cytochrome C oxidase Subunit 4 isoform 1, *SOD2* Superoxide dismutase 2, *GPx1* glutathione peroxidase 1, *BDNF* brain-derived neurotrophic factor, *NF-κB* nuclear factor kappa B, *VEGF* vascular endothelial growth factor, *MMP2/9* Matrix metalloproteinases 2 and 9, *TGF-β* transforming growth factor beta, *TNF-α* tumor necrosis factor alpha, *IL-6* Interleukin 6, *PI3K* Phosphoinositide 3-kinase, *eNOS* endothelial nitric oxide synthase, *NO* nitric oxide, *SIRT3* sirtuin 3

### NURR1 (NR4A2)

NURR1 (NR4A2) is an orphan NR that plays crucial roles in neuronal development, dopamine biosynthesis, mitochondrial function, and neuroinflammation. NURR1 is also involved in various disease processes, particularly in neuroblastoma, where it functions as a tumor suppressor, and in PD, where it offers neuroprotective effects. In both contexts, NURR1’s regulation of key signaling pathways can significantly influence disease progression and response to therapy.

#### NURR1 downregulation in neuroblastoma progression

In neuroblastoma, a prevalent pediatric malignancy, NURR1 (NR4A2) functions as a critical tumor suppressor. Its downregulation is consistently associated with aggressive tumor phenotypes, enhanced metastatic potential, and poor clinical outcomes. One of the primary consequences of NURR1 loss is the impairment of the p53 signaling pathway, a central regulator of the DNA damage response. Normally, NURR1 supports p53-mediated transcription of pro-apoptotic genes; thus, its absence diminishes p53-dependent apoptosis, allowing tumor cells to bypass cell death even under chemotherapeutic stress [231].

NURR1 also modulates cell cycle regulation, influencing key checkpoint proteins such as p21 (CDKN1A) and p27 (CDKN1B). Its suppression leads to deregulated cell cycle progression and increased proliferation. In parallel, NURR1 regulates oxidative stress responses by controlling ROS-detoxifying genes, contributing to DNA stability. When NURR1 is silenced, ROS levels accumulate, causing genomic instability while paradoxically promoting tumor adaptation and survival in hostile microenvironments [231].

Additionally, NURR1 downregulation is implicated in the activation of pro-survival signaling pathways, such as the PI3K/Akt, NF-κB, and MAPK/ERK cascades. These pathways lead to the upregulation of anti-apoptotic proteins like Bcl-2 and Mcl-1, which contribute to chemoresistance by inhibiting the mitochondrial apoptotic machinery. NURR1 also indirectly represses these survival pathways, and its absence lifts this repression, further promoting tumor resilience to treatment [19].

In summary, NURR1 exerts tumor-suppressive effects in neuroblastoma through multiple molecular axes: p53-mediated apoptosis, cell cycle checkpoint control, ROS regulation, and repression of pro-survival signaling pathways including PI3K/Akt, NF-κB, and MAPK/ERK. The loss of NURR1 disrupts these processes, leading to enhanced tumor progression and chemoresistance. Therefore, targeting the NURR1 regulatory network may offer a novel therapeutic strategy for high-risk neuroblastoma.

#### NURR1 activation in Parkinson’s disease (PD)

In PD, NURR1 (NR4A2) plays a crucial neuroprotective role, particularly in dopaminergic neurons of the substantia nigra, where its loss leads to dopaminergic neuron degeneration and the hallmark motor and cognitive symptoms of PD. NURR1 regulates essential processes such as dopamine synthesis, mitochondrial function, and neuroinflammation, which are disrupted in PD. One of the key mechanisms of NURR1’s neuroprotection is the suppression of the NF-κB signaling pathway, which drives neuroinflammation. In PD, chronic NF-κB activation in microglia and astrocytes promotes the release of pro-inflammatory cytokines like TNF-α, IL-1β, and IL-6, exacerbating neuronal injury. NURR1 inhibits NF-κB activity by recruiting CoREST corepressor complexes (including HDAC1, LSD1, and G9a) to NF-κB target gene promoters, repressing cytokine transcription and reducing neurotoxicity [68].

In addition to modulating inflammation, NURR1 mitigates oxidative stress, a key feature of PD pathology, by upregulating antioxidant response genes and maintaining redox balance, protecting neurons from ROS-induced damage. NURR1 also regulates mitochondrial function through the PGC-1α–NURR1–NRF1 pathway, promoting mitochondrial biogenesis and energy production, both of which are compromised in PD. This pathway is crucial for maintaining neuronal energy homeostasis and protecting dopaminergic neurons from mitochondrial dysfunction, a hallmark of the disease [232].

Moreover, NURR1 directly influences dopaminergic gene expression—including tyrosine hydroxylase, aromatic L-amino acid decarboxylase, and dopamine transporter—which are essential for dopamine synthesis, metabolism, and transport. By regulating these genes, NURR1 helps sustain dopaminergic function, supporting motor and cognitive processes impaired in PD [233].

Therapeutically, activating NURR1 holds great promise in PD. Small-molecule NURR1 agonists, such as amodiaquine and C-DIM12, have been shown to reduce neuroinflammation, restore dopamine synthesis, and improve motor function in PD models. These compounds activate NURR1 through modulation of its interactions with coactivators like SRC-1 and PGC-1α and through PTMs, bypassing the need for classical ligand activation. Clinical trials are exploring these agents for their potential to ameliorate PD symptoms in humans [234].

#### Conclusion

NURR1 (NR4A2) serves as a critical regulatory factor in both neuroblastoma progression and Parkinson’s disease. In neuroblastoma, its downregulation contributes to increased tumor aggressiveness, metastasis, and chemoresistance, while in PD, its activation provides neuroprotective effects by promoting mitochondrial function, suppressing neuroinflammation, and preserving dopaminergic neurons. The dual role of NURR1 in these diseases presents an exciting therapeutic opportunity. Developing targeted therapies that either restore or activate NURR1 could lead to novel treatment strategies for high-risk neuroblastoma and PD, offering hope for improving patient outcomes and providing new avenues for drug discovery (Table 4).

**Table 4:** Comparative analysis of NURR1 (NR4A2) roles in neuroblastoma and Parkinson’s disease

Feature	NURR1 in Neuroblastoma	NURR1 in Parkinson's Disease
Role	Tumor suppressor in neuroblastoma	Neuroprotective in Parkinson's disease
Disease	Pediatric malignancy (neuroblastoma)	Neurodegenerative disorder (Parkinson's disease)
Mechanism of action	Suppresses cell proliferation, metastasis, and chemoresistance through various signaling pathways	Protects dopaminergic neurons by modulating inflammation, oxidative stress, and mitochondrial function
Key signaling pathways	p53-mediated apoptosis, PI3K/Akt, NF-κB, MAPK/ERK pathways, oxidative stress regulation	NF-κB, PGC-1α–NURR1–NRF1 (mitochondrial function), dopamine synthesis genes regulation
Gene regulation	Controls transcription of pro-apoptotic genes, oxidative stress response genes, and epigenetic regulation of oncogenes	Regulates dopaminergic genes (TH, AADC, DAT), antioxidant genes, mitochondrial biogenesis
Key effects	Increases tumor aggressiveness, metastasis, and chemoresistance by disrupting cell cycle, apoptosis, and oxidative stress	Reduces neuroinflammation, oxidative damage, and enhances mitochondrial function in dopaminergic neurons
Therapeutic potential	Targeting NURR1 regulatory network to treat high-risk neuroblastoma	NURR1 activation as a therapeutic strategy for PD, including small-molecule agonists (e.g., amodiaquine, C-DIM12)
Epigenetic modulation	Involves HDACs and DNA methyltransferases (DNMTs) for chromatin remodeling and oncogene repression	Recruits corepressor complexes (e.g., CoREST, HDAC1, LSD1, G9a) to suppress pro-inflammatory cytokines in neurodegeneration
Outcome of downregulation	Promotes tumor progression, increased ROS, and resistance to chemotherapy	Leads to dopaminergic neuron degeneration, exacerbating PD symptoms
Outcome of activation	Not applicable (loss of function contributes to malignancy)	Protects neurons, reduces inflammation, restores dopamine synthesis and transport

*NURR1* nuclear receptor-related 1 protein, *NR4A2* nuclear receptor Subfamily 4, group A, member 2, *PD* Parkinson's disease, *ROS* reactive oxygen species, *p53* Tumor protein p53, *p21 (CDKN1A)* cyclin-dependent kinase inhibitor 1A, *p27* *(CDKN1B)* cyclin-dependent kinase inhibitor 1B, *HDACs* histone deacetylases, *DNMTs* DNA methyltransferases, *PI3K/Akt* phosphoinositide 3-kinase/protein kinase B, *NF-κB* nuclear factor kappa-light-chain-enhancer of activated B cells, *MAPK/ERK* mitogen-activated protein kinase/extracellular signal-regulated kinase, *PGC-1α* peroxisome proliferator-activated receptor γ coactivator 1-alpha, *NRF1* nuclear respiratory factor 1, *TH* tyrosine hydroxylase, *AADC* aromatic L-amino acid decarboxylase, *DAT* dopamine transporter, *CoREST* corepressor of REST

### Emerging therapeutic strategies

Recent advancements in NR biology have led to novel treatment strategies for metabolic diseases, cancer, neurodegeneration, and inflammation. Traditional therapies often face challenges, such as off-target activation and metabolic side effects. To overcome these limitations, researchers are focusing on the development of SNRMs, dual and pan-NR agonists, and gene therapies. These emerging approaches aim to refine receptor activation, improve therapeutic efficacy, and minimize adverse effects, advancing the field of precision medicine.

#### Selective nuclear receptor modulators (SNRMs)

Selective SNRMs offer targeted activation of specific NRs, providing therapeutic benefits while minimizing systemic side effects. Unlike full agonists, which activate a broad range of receptor pathways, SNRMs selectively modulate specific NR pathways, making them a promising approach for treating various diseases. Some notable examples of SNRMs include SPPARMs, which modulate insulin sensitivity in T2DM without promoting excessive adipogenesis, a common side effect associated with other PPARγ agonists [235]. sFXRMs improve liver health by modulating bile acid metabolism and enhancing lipid profiles without causing dyslipidemia, a typical concern with broader FXR activation [236]. Tissue-specific SNRMs, such as liver-targeted THR-β agonists like resmetirom, promote hepatic fat oxidation and show promise in treating MASH by reducing liver fat content without affecting other tissues. Resmetirom selectively activates the THR-β receptor in the liver, avoiding systemic effects on tissues like the heart or bones. This liver-specific action enhances lipid metabolism, reduces hepatic steatosis, the core feature of MASH, and promotes fat oxidation. Additionally, resmetirom has shown potential in improving liver function and enzymes, Suggesting benefits in reducing liver inflammation and fibrosis, which are common complications of MaSH. In 2024, resmetirom received FDA approval for MASH, based on clinical trial data showing significant reductions in liver fat and improvements in biomarkers of liver inflammation and damage. This approval marks a significant advancement in liver-targeted therapies for MASH and NASH [237]. Selective Glucocorticoid Receptor Modulators (GRMs) retain anti-inflammatory properties while minimizing the metabolic side effects often seen with traditional glucocorticoid therapies, with potential applications in autoimmune diseases and neurodegenerative conditions like AD and multiple sclerosis. In cancer treatment, selective GR modulators, such as Cort-113 and CORT125134, may reduce tumor growth while minimizing immunosuppressive effects commonly associated with conventional glucocorticoid treatments [238]. SERMs like tamoxifen and raloxifene have been shown to reduce neuroinflammation in AD, while natural compounds like Ginsenoside Rg1 demonstrate protective effects in PD [239].

Additionally, other therapeutic agents like PARP inhibitors (e.g., olaparib, rucaparib) target DNA repair in cancers with BRCA mutations, and HDAC inhibitors (e.g., vorinostat, romidepsin) show promise in inhibiting tumor growth by promoting chromatin relaxation and activating tumor suppressor genes [240]. For cardiovascular protection, PPAR agonists (e.g., pioglitazone, fenofibrate) improve lipid profiles and reduce inflammation, while mineralocorticoid receptor antagonists (e.g., Eplerenone) help prevent heart failure [241]. GLP-1 receptor agonists (e.g., Liraglutide, Semaglutide) and SGLT2 inhibitors (e.g., Empagliflozin, Canagliflozin) improve glucose control and offer cardiovascular protection in T2DM [242]. In Liver disease, seladelpar and elafibranor, both FDA-approved in 2024, are important PPAR agonists for the treatment of PBC. These therapies represent significant advancements in the treatment of liver disorders and further expand the therapeutic potential of PPAR agonists in both metabolic and liver-related conditions. Additionally, SARMs promote muscle growth without the side effects of anabolic steroids, and selective THR modulators, such as sobetirome, provide targeted treatments for metabolic diseases and cancer [243]. While SNRMs represent a significant advancement in targeted therapies, challenges persist regarding their off-target effects. Despite their selectivity, there remains a risk of unintended interactions with other receptors or proteins, which could lead to adverse effects. Future research will focus on enhancing receptor specificity to mitigate these concerns and improve the overall safety and efficacy of SNRMs in clinical applications [244] (Table 5).

**Table 5:** Comparison of selective nuclear receptor modulators (SNRMs) in targeted therapeutic approaches

Therapeutic agent	Target receptor	Therapeutic benefits	Examples	Challenges
Selective PPARγ modulators (SPPARMs)	PPARγ	Modulate insulin sensitivity in Type 2 diabetes without promoting excessive adipogenesis	Pioglitazone, Fenofibrate	Risk of adipogenesis and weight gain
Selective FXR modulators (sFXRMs)	FXR	Improve liver health, modulate bile acid metabolism, enhance lipid profiles without dyslipidemia	OCA Tropifexor	Risk of off-target effects and dyslipidemia
Tissue-specific SNRMs (e.g., Resmetirom)	THR-β (Liver-targeted)	Promote hepatic fat oxidation and reduce liver fat content in NASH without affecting other tissues	Resmetirom	Possible hepatic and off-target effects
Selective glucocorticoid receptor modulators (GRMs)	Glucocorticoid receptor	Retain anti-inflammatory properties while minimizing metabolic side effects in autoimmune and neurodegenerative diseases	Cort-113, CORT125134	Metabolic side effects, immunosuppressive risks
Selective estrogen receptor modulators	Estrogen receptor	Reduce neuroinflammation in Alzheimer’s disease and have protective effects in Parkinson disease	Tamoxifen, Raloxifene	Endometrial and breast cancer risks, thromboembolic events
PARP inhibitors	DNA repair enzymes (PARP)	Target DNA repair in cancers with BRCA mutations, promoting cancer cell death	Olaparib, Rucaparib	Resistance and off-target effects
HDAC Inhibitors	Histone deacetylases	Inhibit tumor growth by promoting chromatin relaxation and activating tumor suppressor genes	Vorinostat, Romidepsin	Potential for toxicity and non-specific effects
PPAR agonists	PPAR	Improve lipid profiles and reduce inflammation in cardiovascular protection	Pioglitazone, Fenofibrate	Risk of off-target side effects, may worsen other conditions
Mineralocorticoid receptor antagonists	Mineralocorticoid receptor	Prevent heart failure by antagonizing mineralocorticoid receptors	Eplerenone	Electrolyte disturbances, risk of hyperkalemia
GLP-1 receptor agonists	GLP-1	Improve glucose control and offer cardiovascular protection in Type 2 diabetes	Liraglutide, Semaglutide	Risk of gastrointestinal side effects, pancreatitis
SGLT2 inhibitors	SGLT2	Improve muscle growth without the side effects of anabolic steroids	Empagliflozin, Canagliflozin	Potential for liver toxicity and kidney issues
SARMs	Androgen receptor	Provide targeted treatments for metabolic diseases and cancer, with fewer systemic side effects	Ostarine, Andarine	Androgenic side effects, liver toxicity
Selective thyroid hormone receptor modulators	THR	Provide targeted treatments for metabolic diseases and cancer, with fewer systemic side effects	Sobetirome	Risk of thyroid dysfunction and off-target effects

*SNRMs* selective nuclear receptor modulators, *SPPARMs* selective PPARγ modulators, *sFXRMs* selective FXR modulators, *THR-β* thyroid hormone receptor beta, *GRMs* selective glucocorticoid receptor modulators, *SERMs* selective estrogen receptor modulators, *PARP* poly(ADP-Ribose) polymerase, *HDAC* histone deacetylases, *PPAR* peroxisome proliferator-activated receptor, *GLP-1* glucagon-like peptide 1, *SGLT2* sodium-glucose co-transporter 2, *SARMs* selective androgen receptor modulators, *OCA* obeticholic acid

#### Dual and pan-nuclear receptor agonists

Dual and pan-NR agonists are emerging therapeutic agents that activate multiple NRs simultaneously, enhancing efficacy by targeting diverse pathways. This approach helps overcome the limitations of therapies targeting a single receptor by enabling more comprehensive metabolic regulation [245]. Notable examples of dual and pan-NR agonists include dual PPARα/γ agonists, such as saroglitazar and NET-144, which improve lipid profiles and insulin sensitivity, offering therapeutic benefits for conditions like T2DM, metabolic syndrome, and MASLD. PPARα/δ agonists enhance mitochondrial function and fatty acid oxidation, making them potential treatments for obesity and muscle disorders, including muscular dystrophy and sarcopenia [246]. Dual FXR/TGR5 agonists target bile acid metabolism and energy regulation pathways, providing benefits for liver fibrosis, thermogenesis, and glucose homeostasis. Pan-NR agonists, such as RGX-202, activate PPARα, PPARγ, and PPARδ, leading to improvements in lipid metabolism, insulin sensitivity, and fatty acid oxidation, with potential applications for metabolic syndrome, dyslipidemia, and CVD [247].

Co-agonists targeting THR and PPARs improve lipid metabolism and reduce cholesterol, offering dual benefits for metabolic diseases. LXR/FXR co-agonists are particularly promising in balancing cholesterol metabolism and bile acid homeostasis, potentially reducing cardiovascular risks [248]. Dual GR/mineralocorticoid receptor agonists modulate inflammation and fibrosis, providing therapeutic options for chronic conditions like kidney disease and heart failure [249]. The development of newer agents, such as PPARδ agonists (e.g., GW0742), which interact with the VDR, enhances glycolysis and fatty acid oxidation, benefiting both metabolic and cardiovascular health. Furthermore, non-secosteroidal VDR agonists are being investigated to provide therapeutic effects without the typical side effects associated with traditional VDR agonists [250].

Although dual and pan-NR agonists show great promise, their multitarget activation could lead to unforeseen side effects. Therefore, further studies are needed to balance the benefits of simultaneous activation with the potential risks of broad receptor engagement (Table 6).

**Table 6:** Comprehensive overview of dual and pan-nuclear receptor agonists in targeted therapeutic approaches

Therapeutic agent	Target receptors	Therapeutic benefits	Examples	Challenges
Dual PPARα/γ agonists	PPARα, PPARβ	Improve Lipid profiles and insulin sensitivity for Type 2 diabetes, metabolic syndrome, NAFLD	Saroglitazar, NET-144	Risk of off-target effects, including excessive adipogenesis
PPARα/δ agonists	PPARα, PPARδ	Enhance mitochondrial function and fatty acid oxidation for obesity, muscular dystrophy, and sarcopenia	GW0742	Potential side effects related to mitochondrial overload and metabolic imbalances
Dual FXR/TGR5 agonists	FXR, TGR5	Target bile acid metabolism, thermogenesis, and glucose homeostasis for liver fibrosis and metabolic regulation	Dual agonists targeting FXR/TGR5	Unintended effects due to broad activation of bile acid metabolism and thermogenesis
Pan-NR agonists	PPARα, PPARβ, PPARγ	Activate multiple PPARs to improve lipid metabolism, insulin sensitivity, and fatty acid oxidation for metabolic diseases	RGX-202	Risk of broad receptor engagement leads to side effects and imbalances
Co-agonists (THR and PPARs)	THR, PPARs	Improve lipid metabolism and reduce cholesterol for metabolic diseases	Co-agonists for THR and PPARs	Possible adverse interactions with thyroid hormone signaling
LXR/FXR Co-agonists	LXR, FXR	Balance cholesterol metabolism and bile acid homeostasis to reduce cardiovascular risks	LXR/FXR co-agonists	Potential imbalance in cholesterol and bile acid homeostasis, leading to side effects
Dual GR/MR agonists	GR, MR	Modulate inflammation and fibrosis in chronic kidney disease and heart failure	Dual GR/MR agonists	Risk of excessive suppression of inflammation and fibrosis modulation
PPARδ agonists interacting with VDR	PPARδ, VDR	Enhance glycolysis and fatty acid oxidation, benefiting metabolic and cardiovascular health	GW0742 and VDR interactions	Side effects from broad activation, particularly in metabolic pathways
Non-secosteroidal vitamin D receptor agonists	VDR	Provide therapeutic effects without typical side effects of traditional VDR agonists	Non-secosteroidal VDR agonists	Possible toxicity and off-target effects, including vitamin D-related side effects

*SNRAs* selective nuclear receptor agonists, *PPAR* peroxisome proliferator-activated receptor, *FXR* farnesoid X receptor, *TGR5—G* protein-coupled bile acid receptor 1, *THR* thyroid hormone receptor, *LXR* liver X receptor, *GR* glucocorticoid receptor, *MR* mineralocorticoid receptor, *VDR* vitamin D receptor, *NAFLD* non-alcoholic fatty liver disease, *GW0742* A PPARδ agonist, *SARs* selective androgen receptor modulators

#### Gene therapy approaches for nuclear receptor dysregulation

Gene therapy strategies for NRs dysregulation are rapidly advancing, offering promising long-term solutions for diseases caused by genetic NR defects. These therapies aim to restore normal receptor function by targeting specific NRs involved in various conditions. Notable examples include HNF4α gene therapy, which seeks to restore insulin secretion in MODY by correcting mutations in the HNF4α gene, crucial for pancreatic β-cell function [251]. AAV-mediated Delivery of NURR1 has shown promise in PD by protecting dopaminergic neurons from degeneration through the delivery of functional NURR1 to affected brain regions [252]. PPARγ gene therapy targets PPARγ to improve insulin sensitivity and lipid metabolism in individuals with metabolic syndrome, offering a potential cure for disorders stemming from PPARγ dysfunction [253]. In cancer gene therapy, CRISPR-based precision gene editing tools are being developed to correct NR mutations contributing to hormone-related cancers, potentially enhancing therapeutic responses and improving patient outcomes [254].

RNA-based therapies, such as siRNA and antisense oligonucleotides, are also being explored to modulate NR expression and splicing, offering non-invasive alternatives to traditional gene therapy [255]. Advances in non-viral delivery systems, including lipid nanoparticles and exosomes, are enabling targeted, tissue-specific modulation of NRs, which could improve the safety and efficiency of these therapies [256]. In addition to genetic interventions, epigenetic editing tools, such as CRISPR/Cas9-based modulation, are being investigated to reprogram NR expression without altering the underlying DNA sequence, providing another layer of therapeutic precision [257]. Coupled with AI-driven drug discovery and personalized medicine, these approaches hold the potential to revolutionize treatments for metabolic, neurodegenerative, and oncological diseases by enabling precise interventions for NR dysregulation [258].

While gene therapies offer tremendous potential, challenges remain particularly in delivery mechanisms, immunogenic responses, and long-term effectiveness. Nonetheless, with continued research and innovation, these therapies could provide lasting solutions to diseases driven by NR dysfunction (Table 7).

**Table 7:** Gene therapy approaches for nuclear receptor dysregulation: promising strategies for targeted interventions

Therapeutic Approach	Target Receptor	Therapeutic Benefits	Examples	Challenges
HNF4α gene therapy	HNF4α	Restores insulin secretion in monogenic diabetes (MODY) by correcting HNF4α mutations	Gene therapy targeting HNF4α in MODY patients	Delivery mechanisms and efficiency in pancreatic β-cells
AAV-mediated delivery of NURR1	NURR1	Protects dopaminergic neurons in Parkinson’s disease by delivering functional NURR1 to the brain	AAV-based NURR1 delivery for Parkinson's disease	Difficulty in delivering AAV to specific brain regions and avoiding immune responses
PPARÎ^3^ gene therapy	PPARÎ^3^	Improves insulin sensitivity, lipid metabolism in individuals with metabolic syndrome	PPARÎ^3^ gene therapy for metabolic syndrome	Long-term effectiveness and potential side effects in metabolic tissues
Cancer gene therapy (CRISPR-based)	NRs in hormone-related cancers	Corrects NR mutations contributing to hormone-related cancers, enhancing therapeutic response	CRISPR/Cas9-based gene editing in hormone-related cancers	Off-target effects and precision in targeting NR mutations in cancer cells
siRNA-based therapies	Various NRs	Modulates NR expression and splicing using RNA interference, offering non-invasive treatment options	siRNA-based therapies for NR modulation	Efficient delivery and stability of siRNA in tissues
Antisense oligonucleotides	Various NRs	Regulates NR expression by binding to RNA, providing precision therapy for various diseases	Antisense oligonucleotides targeting NR splicing	Off-target effects and ensuring specificity in NR splicing modulation
Non-viral Delivery Sys (Lipid NPs, Exosomes)	Various NRs	Improves safety and efficiency of gene delivery, enabling tissue-specific modulation of NRs	Lipid nanoparticles, exosomes for NR delivery	Immunogenic responses to non-viral delivery vehicles
Epigenetic editing (CRISPR/Cas9-based)	Various NRs	Enables targeted modulation of NR expression without altering the DNA sequence, providing precise interventions	CRISPR/Cas9 epigenetic modulation of NR expression	Off-target effects and long-term safety of epigenetic interventions
AI-driven drug discovery and personalized medicine	Various NRs	Uses AI to identify drug candidates and personalize treatments for diseases involving NR dysregulation	AI-driven personalized therapies targeting NR pathways	Complexity of AI-driven drug discovery and integrating personalized medicine

*NR* nuclear receptor, *HNF4α* Hepatocyte nuclear factor 4 alpha, *AAV* Adeno-associated virus, *NURR1* nucleus accumbens gene 1, *PPARγ* peroxisome proliferator-activated receptor gamma, *CRISPR* clustered regularly interspaced short palindromic repeats, *siRNA* small interfering RNA, *ASOs* antisense oligonucleotides, *VDR* vitamin D receptor, *MODY* maturity-onset diabetes of the young, *AI* artificial intelligence, *RNA* ribonucleic acid

### Challenges in nuclear receptor-targeted therapies

While NR-targeted therapies have made notable progress, several challenges remain in their clinical application and long-term effectiveness. A major hurdle is achieving tissue-specific activation to avoid off-target effects. Because NRs are pleiotropic, their activation can yield beneficial outcomes in one tissue while causing unintended adverse effects in another [59]. Resistance mechanisms—especially in chronic diseases like cancer, metabolic disorders, and cardiovascular conditions—can undermine the effectiveness of NR-based therapies over time. Moreover, balancing the metabolic benefits of NR activation with potential carcinogenic risks remains a critical concern. Ongoing research is focused on developing tissue-selective modulators, overcoming resistance, and ensuring long-term safety, which will be key to enhancing the therapeutic potential of NR-targeted treatments.

#### Tissue-specific activation to reduce side effects

The tissue-specific activation of NRs is essential for maximizing therapeutic benefits while minimizing adverse effects. For example, PPARγ agonists like TZDs not only improve insulin sensitivity in adipose tissue but can also lead to weight gain and fluid retention due to off-target activation in other organs such as the liver and kidneys [259]. Similarly, FXR agonists like OCA are beneficial in reducing liver fibrosis in MASH, but their broader effects, including raising LDL cholesterol and causing pruritus, can complicate treatment [120].

To overcome these limitations, researchers are developing tissue-selective SNRMs that specifically activate NR signaling in target organs. For instance, SPPARMs enhance insulin sensitivity without triggering excessive adipogenesis, thus reducing the risk of weight gain and fluid retention. Similarly, liver-specific thyroid hormone receptor (THR-β) agonists like resmetirom aim to target hepatic lipid metabolism in the treatment of MASLD and MASH, without influencing cardiovascular function. Likewise, tissue-specific GR modulators retain anti-inflammatory benefits while minimizing side effects such as weight gain and insulin resistance.

Advancements in ligand engineering, structural biology, and AI-driven drug discovery are enhancing the specificity of NR modulators, allowing for the selective activation of beneficial pathways while avoiding unwanted effects. Furthermore, novel drug delivery systems, including nanoparticles and tissue-specific receptor ligands, are being explored to enhance selectivity and efficacy, ensuring that only the targeted tissue or organ is affected (Table 8).

**Table 8:** Optimizing therapeutic efficacy with tissue-specific activation of nuclear receptors

Therapeutic approach	Target receptors	Therapeutic benefits	Examples	Challenges
PPARγ agonists (e.g., TZDs)	PPARγ	Improves insulin sensitivity in adipose tissue but can cause weight gain and fluid retention in other organs	TZDs, Rosiglitazone, Pioglitazone	Off-target activation in organs like the liver and kidneys, leading to weight gain and fluid retention
FXR agonists (e.g., obeticholic acid)	FXR	Reduces liver fibrosis in NASH but can raise LDL cholesterol and cause pruritus	Obeticholic Acid (OCA)	Potential for raising LDL cholesterol and causing pruritus, complicating treatment
Selective PPARγ modulators (SPPARMs)	PPARγ	Enhances insulin sensitivity without triggering excessive adipogenesis, thus reducing weight gain and fluid retention	Selective PPARÎ^3^ modulators (SPPARMs)	Ensuring tissue-specific activation without affecting other tissues, reducing side effects
Liver-Specific THR-β Agonists (e.g., Resmetirom)	THR-β	Targets hepatic lipid metabolism for NAFLD and NASH treatment, avoiding cardiovascular effects	Resmetirom	Maintaining liver-specific targeting without affecting other systems like cardiovascular function
Tissue-specific glucocorticoid receptor (GR) modulators	GR	Retains anti-inflammatory benefits while minimizing side effects such as weight gain and insulin resistance	Tissue-specific GR modulators	Minimizing side effects like weight gain and insulin resistance while maintaining anti-inflammatory effects

*PPARγ* peroxisome proliferator-activated receptor gamma, *FXR* farnesoid X receptor, *THR-β* thyroid hormone receptor beta, *GR* glucocorticoid receptor, *NASH* non-alcoholic steatohepatitis, *TZDs* thiazolidinediones, *OCA* obeticholic acid, *SPPARMs* selective PPARγ modulators, *NAFLD* non-alcoholic fatty liver disease, *LDL* low-density lipoprotein

#### Resistance mechanisms in chronic diseases

Resistance mechanisms in chronic diseases Such as type 2 diabetes, MASH, cancer, and cardiovascular disorders present significant challenges to the sustained effectiveness of NR-targeted therapies. Chronic NR activation can lead to disease adaptation, where the body’s metabolic pathways adjust over time, diminishing the drug's responsiveness. This adaptation often triggers compensatory mechanisms that exacerbate metabolic dysregulation or reduce the therapeutic effects.

For instance, prolonged activation of FXR in liver disease can initially improve bile acid homeostasis but may eventually lead to a desensitization of the receptor, reducing the drug's effectiveness and contributing to disease progression. Similarly, long-term use of PPARγ agonists can result in receptor downregulation and secondary insulin resistance, making the treatment less effective over time [260]. In the context of cancer, tumor cells may circumvent hormone-targeted therapies by activating alternative survival pathways, a phenomenon often referred to as therapeutic resistance [261].

To address these challenges, researchers are developing next-generation NR modulators that target multiple receptor pathways and are exploring combination therapies with metabolic, epigenetic, or immune modulators to overcome these adaptive mechanisms. Additionally, advances in single-cell transcriptomics and metabolic profiling are helping identify early biomarkers of resistance, enabling more personalized NR-targeted interventions that are tailored to individual disease progression (Table 9).

**Table 9:** Addressing resistance mechanisms in chronic diseases: overcoming challenges in NR-targeted therapies

Therapeutic challenge	Target receptors	Challenges	Potential solutions	Examples	Challenges in implementation
FXR activation in liver disease	FXR	Prolonged FXR activation leads to receptor desensitization, reducing therapeutic effects and contributing to disease progression in liver disease	Development of next-generation FXR modulators targeting compensatory pathways in liver disease and enhancing receptor selectivity	OCA for liver disease, next-generation FXR modulators	Risk of non-specific activation, potential off-target effects in tissues, and insufficient receptor specificity
PPARγ agonists in metabolic disease	PPARγ	Long-term use of PPARγ agonists can result in receptor downregulation, insulin resistance, and reduced effectiveness in metabolic disease treatment	Combination therapies with alternative metabolic modulators and newer PPARÎ^3^ modulators to reduce insulin resistance	Pioglitazone, Rosiglitazone, newer PPARγ agonists	Difficulty in maintaining effective long-term insulin sensitivity without triggering side effects like weight gain and fluid retention
Resistance mechanisms in cancer	Various hormone receptors	Tumor cells activate alternative survival pathways, circumventing hormone-targeted therapies, leading to therapeutic resistance in cancer	Targeting alternative survival pathways in tumor cells, combining NR modulators with immune or epigenetic therapies to overcome resistance	Combination therapies in cancer with immune modulators, epigenetic agents, and NR-targeted therapies	Tumor heterogeneity and lack of effective biomarkers to identify specific resistance mechanisms across different cancer types
Resistance mechanisms in cardiovascular disease	Various NR receptors	Chronic activation of NR-targeted therapies in CVs leads to resistance, with compensatory mechanisms undermining drug efficacy over time	Exploring NR combination therapies with immune, metabolic, and epigenetic modulators to improve cardiovascular outcomes	Combination NR-targeted therapies for cardiovascular diseases, new drug formulations targeting compensatory mechanisms	Risk of chronic drug resistance and difficulty in achieving sustained effects with single-target therapies in cardiovascular diseases
Adaptive resistance in chronic diseases	Multiple NRs	Chronic diseases exhibit adaptive resistance where metabolic pathways adjust, decreasing the responsiveness to NR-targeted therapies	Personalized NR-targeted therapies, aided by single-cell transcriptomics and metabolic profiling to identify early biomarkers of resistance	Single-cell transcriptomics, metabolic profiling, personalized NR therapy	Challenges in identifying biomarkers of resistance and tailoring therapies for individual disease progression in chronic conditions

*FXR* farnesoid X receptor, *PPARγ* peroxisome proliferator-activated receptor gamma, *NR* nuclear receptor, *OCA* obeticholic acid, *NASH* non-alcoholic steatohepatitis, *TZDs* thiazolidinediones, *AI* artificial intelligence, *MODY* maturity-onset diabetes of the young

#### Balancing metabolic benefits with potential carcinogenic risks

While NR agonists and modulators provide significant metabolic benefits, their potential carcinogenic risks raise concerns about long-term safety. Chronic or systemic activation of NRs can inadvertently promote tumorigenesis in certain tissues. For example, PPARγ activation has been associated with an increased risk of bladder cancer [262], and prolonged LXR activation has been linked to liver tumor formation in animal models. Similarly, FXR modulators have been implicated in an increased risk of HCC [263].

To mitigate these risks, researchers are developing NR modulators with improved safety profiles, including partial agonists and inverse agonists that selectively activate beneficial pathways while minimizing unwanted effects. Strategies such as short-term activation, intermittent dosing, and tissue-specific targeting are also being explored to limit prolonged NR activation. Additionally, CRISPR-based gene editing and genome-wide association studies are being employed to identify high-risk populations, allowing for more personalized risk assessments and preventive strategies.

In conclusion, while NR-targeted therapies hold tremendous promise, significant challenges remain, particularly in terms of tissue-specific activation, resistance mechanisms, and carcinogenic risks. The development of next-generation SNRMs, combination therapies, and personalized treatment approaches will be critical in enhancing both the efficacy and safety of these therapies, ultimately ensuring long-term disease management.

## Research gaps and future prospects

NR-targeted therapies offer immense promise in treating a variety of diseases, from metabolic disorders to cancer and neurodegenerative diseases. However, several research gaps and challenges need to be addressed to maximize their therapeutic potential. Key areas of focus include improving tissue-specific modulation, overcoming resistance mechanisms, and addressing safety concerns, particularly with respect to carcinogenic risks. To make NR-targeted therapies clinically viable and effective for long-term use, advancements in drug delivery, gene therapy, and precision medicine are essential.

### Tissue-specific modulation of nuclear receptors

One of the most pressing challenges in the development of NR-targeted therapies is the lack of tissue-specific modulation. Most pharmacological agents currently available activate NRs across multiple tissues, leading to undesirable systemic side effects. For instance, LXR agonists can induce lipogenic activity in the liver, while PPARγ agonists can cause fluid retention and weight gain in adipose tissue. These side effects arise due to the broad activation of NRs in non-target tissues.

To address these issues, future research must focus on developing highly SNRMs that can target specific tissues affected by disease. Advances in ligand design, structural biology, and AI-driven drug discovery hold the potential to refine NR therapies, improving both safety and efficacy. Furthermore, advanced drug delivery systems, such as nanoparticles or tissue-specific receptor ligands, could enable more precise targeting of tissues, thereby reducing off-target effects and improving therapeutic outcomes.

### Resistance mechanisms in chronic diseases

In chronic diseases like T2DM, MASLD, CVDs, and hormone-dependent cancers, prolonged NR activation often triggers adaptive responses that lead to resistance. Over time, this resistance manifests as receptor desensitization, altered gene expression, and diminished drug efficacy. For example, prolonged use of PPARγ agonists can lead to receptor downregulation, resulting in secondary insulin resistance.

To combat this, future strategies should aim at dual or multi-receptor modulation, combining metabolic and epigenetic regulators to enhance therapeutic efficacy and prevent resistance. Intermittent activation approaches, where NR activation is alternated with periods of inactivity, may also help in maintaining the efficacy of NR therapies. Moreover, single-cell transcriptomics and metabolic profiling techniques could help identify early biomarkers of resistance, enabling the development of more personalized treatment strategies based on disease progression.

### Clinical translation and standardization challenges

Despite promising preclinical results, translating NR-based therapies into clinical practice has proven challenging. Barriers include a lack of standardized dosing regimens, insufficient long-term safety data, and the need for better patient-specific response profiling. The variability in individual responses to NR-targeted therapies further complicates treatment optimization.

To address these challenges, large-scale, well-controlled randomized clinical trials (RCTs) are essential to establish the optimal dosing, safety, and efficacy of NR-based therapies across diverse patient populations. Additionally, biomarker-driven patient stratification can enable the development of precision medicine approaches, where treatments are tailored based on genetic, metabolic, and disease-specific factors, potentially improving treatment outcomes.

### Nuclear receptors and gut microbiome interactions

A new and exciting area of research is the interaction between NRs and the gut microbiome, particularly in regulating bile acid metabolism, immune function, and metabolic homeostasis. NRs such as FXR, LXR, and PPARs are intricately involved in the composition of gut microbiota, influencing the development of metabolic disorders, inflammatory diseases, and liver conditions.

While the precise mechanisms by which microbiome-derived metabolites modulate NR activity remain unclear, emerging research indicates that microbiome-targeted therapies could become a valuable tool in managing NR-driven diseases. Future studies integrating metagenomics, metabolomics, and NR-specific modulators will be critical in developing innovative therapeutic strategies. These could include dietary modifications, probiotics, or even microbiome-derived NR modulators to enhance the management of diseases like MASH and metabolic syndrome.

### Neuroprotective potential of nuclear receptors

The potential of NRs in the treatment of neurodegenerative diseases, such as AD and PD, has gained significant attention. PPARγ agonists, NURR1 activators, and FXR modulators have shown promising neuroprotective effects in preclinical studies. However, the mechanisms by which NRs influence neurodegeneration, synaptic plasticity, and cognitive function remain inadequately understood.

Barriers such as BBB permeability, neuronal specificity, and concerns over long-term safety must be addressed before NR-targeted therapies can be applied in the CNS. The development of nanotechnology-based drug delivery systems, gene therapy, and neural-targeted SNRMs may help overcome these challenges, making NR-based neuroprotective strategies more feasible.

### Gene therapy for nuclear receptor dysregulation

Gene therapy holds great promise for treating diseases caused by NR dysregulation. Approaches such as CRISPR-based gene editing, RNA therapeutics, and epigenetic modulation are showing potential in correcting NR dysfunction at the genomic level. However, challenges such as vector safety, gene-editing specificity, and the long-term stability of therapeutic effects still need to be addressed.

Future research should focus on refining gene delivery methods, improving control over gene expression, and enhancing precision in gene-editing techniques to bring NR modulation-based gene therapies closer to clinical application. These advancements could have significant implications for treating metabolic disorders, neurodegeneration, and cancer by reprogramming NR-mediated pathways.

### Carcinogenic risks and long-term safety concerns

A major concern in NR-targeted therapies is the potential for carcinogenic risks associated with prolonged activation of certain NRs. For instance, PPARγ, FXR, and LXR agonists have been linked to tumorigenesis in hormone-sensitive cancers and liver diseases. Chronic NR activation can influence cell proliferation, oncogenic signaling, and interactions within the tumor microenvironment, raising concerns about sustained drug exposure.

To mitigate these risks, future strategies should focus on developing partial agonists, inverse agonists, and intermittent dosing regimens that preserve therapeutic efficacy while minimizing carcinogenic risks. Integrating multi-omics analysis, AI-driven predictive modeling, and precision medicine approaches could further optimize the safety and effectiveness of NR-targeted therapies.

### Conclusion

While NR-based therapies hold substantial promise for treating a wide range of diseases, several challenges remain in optimizing their clinical use. Tissue-specific modulation, overcoming resistance mechanisms, addressing clinical translation hurdles, and mitigating carcinogenic risks are key areas of focus. By embracing precision medicine, advancing drug delivery technologies, and leveraging AI-driven drug discovery, the full therapeutic potential of NR modulation can be realized. These advancements could transform the treatment of complex diseases and improve patient outcomes, paving the way for a new era in molecular medicine.

## Study limitations

### Lack of large-scale clinical trials

A significant limitation in the development of NR-based therapies is the scarcity of large-scale, randomized controlled trials (RCTs) that include diverse patient populations. While numerous NR agonists and modulators have demonstrated promising results in preclinical models, their clinical translation remains limited. The absence of comprehensive, well-controlled human trials leaves key aspects, such as long-term safety, efficacy, and optimal dosing strategies, uncertain. This uncertainty not only hampers regulatory approval but also delays the widespread clinical adoption of NR-based therapies. To overcome this limitation, future studies must prioritize large-scale RCTs that better reflect the heterogeneity of patient populations, including varying ages, genetic backgrounds, and comorbid conditions.

### Underexplored nuclear receptors and crosstalk mechanisms

While this review has primarily focused on well-characterized NRs such as PPARs, FXR, LXR, TR, and members of the NR4A family, many lesser-known or orphan NRs have yet to be fully explored, despite their potential therapeutic promise. Receptors like RORα and PXR hold untapped potential, but research into their endogenous ligands, mechanisms of action, and pharmacological applications remains limited. Moreover, the crosstalk between NRs and other cellular pathways—including interactions with the gut microbiome, immune system, and epigenetic modifications—is another largely unexplored area. These interconnections could significantly influence NR activity and therapeutic outcomes, making it imperative for future research to investigate these pathways in more detail to unlock new therapeutic avenues.

### Limited clinical validation of emerging therapeutic strategies

Emerging strategies, such as selective SNRMs, dual and pan-NR agonists, and gene therapy, remain largely experimental and have not undergone extensive clinical validation. While these next-generation therapies hold promise for improving tissue specificity, reducing side effects, and overcoming drug resistance, their clinical application faces significant challenges. The lack of standardized evaluation methods, safety assessments, and regulatory approvals has hindered their progression into clinical practice. For example, SNRMs show potential for targeted NR modulation in tissues like the liver or adipose tissue, but their therapeutic benefits and risks are still not fully understood in human trials. To bridge the gap between preclinical research and clinical applications, it is critical to develop standardized evaluation methods and conduct comprehensive safety and efficacy assessments. Precision medicine strategies, such as patient-specific drug profiles and biomarkers, will be crucial for ensuring the safe and effective application of these therapies in clinical settings.

### Summary

NRs are a diverse family of ligand-activated transcription factors that regulate metabolism, inflammation, cardiovascular health, neurodegeneration, and cancer. By modulating gene expression in response to metabolic and hormonal cues, they hold major therapeutic potential for chronic diseases. Key members—such as PPARs, FXR, LXR, TR, HNF4α, and NR4A—govern lipid and glucose metabolism, bile acid signaling, mitochondrial function, and immune responses.

Approved and investigational therapies include PPARγ agonists (pioglitazone) for diabetes, FXR agonists (OCA) for liver disease, LXR modulators for atherosclerosis, and THR-β agonists (resmetirom) for MASH. Despite promising results, challenges remain in achieving tissue-selective activation, overcoming resistance, and ensuring long-term safety, underscoring the need for more selective SNRMs, dual/pan-NR agonists, and precision approaches.

Advances in AI-driven drug discovery, gene therapy, and microbiome–NR research are helping address these limitations, while expanding applications in neurodegeneration and immune modulation. Future priorities include optimizing tissue-specific delivery, refining gene-editing tools, and integrating multi-omics for personalized therapy. Addressing these gaps could position NR-based strategies to transform the treatment of metabolic, inflammatory, neurodegenerative, and cancer-related diseases.

## Conclusion

NRs are central regulators of metabolism, inflammation, immune function, and cellular homeostasis, offering major therapeutic potential for metabolic disorders, CVDs, cancer, and neurodegeneration. Despite progress in NR-targeted therapies, clinical translation faces hurdles including tissue specificity, off-target effects, resistance, and long-term safety.

Overcoming these challenges will require precision-targeted therapies and delivery systems that enable tissue-specific activation while minimizing adverse effects. Emerging tools—such as AI-driven drug discovery, CRISPR-based gene editing, and microbiome-based interventions—offer promising solutions. AI can predict tissue-specific actions, CRISPR can fine-tune NR pathways, and biomarker-guided precision medicine can tailor treatments to individual patients.

The therapeutic scope of NRs remains vast, but realizing their full clinical impact will demand multidisciplinary collaboration to close research gaps, refine strategies, and ensure safety and efficacy in long-term use.

## Data Availability

No datasets were generated or analysed during the current study.

## Abbreviations

ABC: ATP-binding cassette
ABCA1: ATP-binding cassette transporter A1
ACOX1: Acyl-CoA oxidase 1
AD: Alzheimer’s disease
AMPK: AMP-activated protein kinase
AP-1: Activator protein-1
AR: Androgen receptor
BBB: Blood–brain barrier
CPT1: Carnitine palmitoyltransferase 1
CVD: Cardiovascular disease
ER: Estrogen receptor
ERRα: Estrogen-related receptor alpha
eNOS: Endothelial nitric oxide synthase
FXR: Farnesoid X receptor
GLUT4: Glucose transporter 4
GR: Glucocorticoid receptor
HDAC: Histone deacetylase
HNF4α: Hepatocyte nuclear factor 4 alpha
HREs: Hormone response elements
IL-1β: Interleukin-1 beta
IL-6: Interleukin-6
IRS-1/IRS-2: Insulin receptor substrates 1 and 2
LDL: Low-density lipoprotein
LXR: Liver X receptor
MAFLD: Metabolic dysfunction-associated fatty liver disease
MAPK: Mitogen-activated protein kinase
MASLD: Metabolic dysfunction-Associated Steatotic Liver Disease
MASH: Metabolic dysfunction-Associated Steatohepatitis
MODY: Maturity-onset diabetes of the young
mTOR: Mammalian target of rapamycin
NF-κB: Nuclear factor kappa B
NO: Nitric oxide
NR: Nuclear receptor
NURR1 (NR4A2): Nuclear receptor-related 1 protein
OCA: Obeticholic acid
PD: Parkinson’s disease
PGC-1α: Peroxisome proliferator-activated receptor gamma coactivator-1 alpha
PBC: Primary biliary cholangitis
PPARα/γ/δ: Peroxisome proliferator-activated receptor alpha/gamma/delta
RXR: Retinoid X receptor
SIRT1: Sirtuin 1
sFXRMs: Selective FXR modulators
SNRMs: Selective nuclear receptor modulators
SPPARMs: Selective PPARγ modulators
T2DM: Type 2 diabetes mellitus
THR: Thyroid hormone receptor
TNF-α: Tumor necrosis factor alpha
TGR5: Takeda G protein-coupled receptor 5
TZDs: Thiazolidinediones
UCP1: Uncoupling protein 1
VDR: Vitamin D receptor
VLDL: Very low-density lipoprotein
